# Thermodynamic stabilization of substrate protein within the GroEL:ES chaperonin cage

**DOI:** 10.1073/pnas.2624029123

**Published:** 2026-11-03

**Authors:** Wei Xue, Andreas Bracher, F. Ulrich Hartl, Manajit Hayer-Hartl

**Affiliations:** ^a^ Department of Cellular Biochemistry, Max Planck Institute of Biochemistry, Martinsried 82152, Germany

**Keywords:** protein folding, chaperonin, GroEL, GroES, maltose-binding protein

## Abstract

The adenosine triphosphate (ATP)-regulated GroEL/ES chaperonin of *Escherichia coli* promotes protein folding by transiently encapsulating unfolded substrate protein in a nano-cage formed by the cylindrical GroEL and the lid-shaped GroES. How this confinement modulates the folding energy landscape, allowing accelerated protein folding unimpaired by aggregation, remains incompletely understood. Prompted by recent observations suggesting that the chaperonin cavity may favor unfolding [I. Korobko *et al.*, *Proc. Natl. Acad. Sci. U.S.A.* **119**, e2213170119 (2022)], here we reinvestigated the effect of encapsulation on protein thermodynamic stability. To allow thermal unfolding of an encapsulated substrate protein, we generated a destabilized variant of maltose-binding protein (MBP) by introducing four mutations, quadruple mutant-MBP (QM-MBP), lowering the melting temperature (*T_m_*) from 63.6 °C to 44.3 °C. GroEL/ES accelerated the slow spontaneous folding of QM-MBP ~20-fold. The protein reached a native-like structure while encapsulated inside GroEL:ES, as demonstrated using hydrogen/deuterium exchange and single molecule fluorescence resonance energy transfer analyses in presence and absence of the ligand maltose. Importantly, confinement in the thermally stable chaperonin shifted the cooperative unfolding transition of QM-MBP to a *T_m_* of 52.4 °C, corresponding to a stabilization by 4.6 kcal mol^−1^. In-cage thermal unfolding was reversible, indicating that the environment of the chaperonin cavity is conducive to folding. These findings suggest that a thermodynamically stabilizing effect of the GroEL:ES cage contributes to promoting protein folding.

## Significance

The GroEL/ES chaperonin system of bacteria promotes protein folding by transiently encapsulating unfolded substrate protein in a nano-cage formed by the cylindrical GroEL and the lid-shaped GroES. Homologous chaperonins function in mitochondria and chloroplasts. While the encapsulation mechanism is well established, how confinement inside the chaperonin cage modulates the energy landscape of protein folding is not yet well understood. Here, we show using a combination of biochemistry and biophysical measurements that the native-like folded state of a mutationally destabilized model protein is markedly thermodynamically stabilized when inside the chaperonin cage. These findings advance our mechanistic understanding of chaperonin-mediated protein folding, an essential process in all domains of life.

The chaperonins are among the most extensively studied molecular chaperones, with GroEL of *E. coli* as a prototype. Close homologs of GroEL are present in mitochondria (Hsp60) and chloroplasts (Cpn60), and more distantly related chaperonins in the cytosol of archaea (thermosome) and eukarya (TRiC/CCT) (1–6). These essential multisubunit complexes with ATPase activity function as nano-cages for a single molecule of substrate protein to fold efficiently, unimpaired by aggregation (3, 5, 7).

**Fig. 1.**
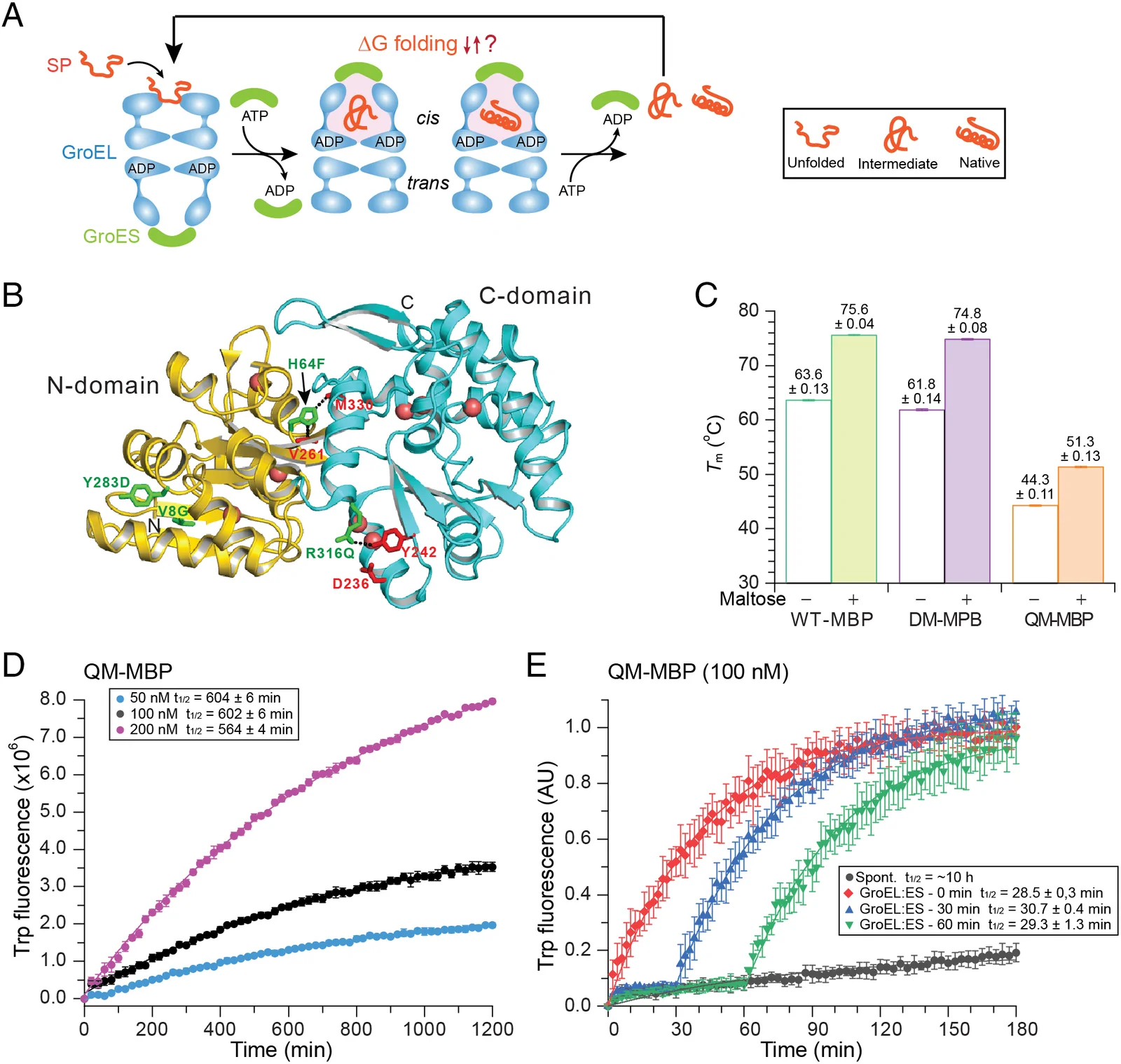
Folding and stability of QM-MBP. (*A*) Simplified scheme for ATP-dependent protein folding by GroEL/ES. Different states of the substrate protein are shown in red. (*B*) Structural model of ligand-free MBP. MPB (PDB: 1OMP) is shown in ribbon representation with N- and C-domains indicated in gold and cyan, respectively. The residue substitutions in the QM-MBP mutant are indicated and the respective residues and their contacts are shown in stick representation in green and red, respectively. Hydrogen bonds are shown as dotted lines. Tryptophan (Trp) residues are indicated as spheres (salmon). N- and C-termini are marked. (*C*) MBP stability. Bar graphs show the melting temperatures (*T*_m_) of WT-, DM-, and QM-MBP in refolding buffer A, in the presence or absence of 10 mM maltose ligand (mean ± SEM, n = 3). (*D*) Spontaneous refolding of QM-MBP. QM-MBP denatured in 6 M GuHCl was diluted 200-fold into refolding buffer A at the final concentrations indicated. The increase of intrinsic Trp fluorescence was monitored over 20 h at 20 °C. Mean values are plotted; error bars represent SEM (n = 3). The half-times of folding (t_1/2_) determined by exponential fitting are indicated. (*E*) Acceleration of QM-MBP refolding by GroEL/ES. GroEL (1 μM), GroES (2 μM), and ATP (5 mM) were added to 100 nM QM-MBP refolding reactions [as in (*D*)] at the indicated time points: 0 min (red), 30 min (blue), and 60 min (green). Spontaneous refolding of QM-MBP is shown as a reference (black).

GroEL is a cylindrical ~800 kDa complex consisting of two heptameric rings of ~57 kDa subunits stacked back-to-back. It cooperates with GroES, a dome-shaped single-ring of seven ~10 kDa subunits (3, 5). The GroEL subunits have a discontinuous domain structure composed of apical, intermediate, and equatorial domains (7). The apical domains form the ring opening, providing hydrophobic binding sites for complementary hydrophobic regions of a nonnative substrate protein. The equatorial domains, mediating intersubunit contacts within and between rings, bind and hydrolyze ATP, with the intermediate, hinge-like domains transmitting nucleotide-dependent conformational changes to the apical domains (3, 7). Client protein binds as a collapsed folding intermediate to an open GroEL ring, experiencing a conformational expansion which may resolve nonnative intramolecular contacts (3, 5, 8). Binding of ATP to the substrate-containing GroEL ring then induces an upward and outward movement of the apical domains so as to allow the lid-shaped GroES to bind, thereby triggering release of bound protein from the apical domains and its encapsulation in a cage-like structure formed by the GroEL ring cavity and the GroES lid (referred to as the *cis* ring) (Fig. 1*A*). During encapsulation, the hydrophobic binding sites on the apical domains become buried, resulting in a mainly hydrophilic and net-negatively charged inner wall of the cage. The enclosed protein (up to ~60 kDa in size) is now free to fold for 2 to 10 s, the time it takes the seven GroEL subunits of the *cis* ring to hydrolyze their bound ATP. The cis ring opens upon ATP binding to the GroEL trans ring through negative interring allostery (3, 5, 9), allowing client protein release and resetting the chaperonin cycle (Fig. 1*A*). Folding may either be completed in a single round or require multiple interaction cycles. GroEL and GroES are essential under all growth conditions (10) and are massively upregulated (5 to 10-fold) upon heat stress, allowing *E. coli* to grow at temperatures up to 50 °C (10, 11).

Extensive biochemical and structural evidence demonstrates that monomeric substrate proteins fold to their native (or a native-like) state inside the chaperonin cage, while protomers of oligomeric proteins reach a folded conformation competent for assembly upon release (3, 5). A recent cryo-EM structure at 3.7 Å resolution shows the MetK protomer, an obligate GroEL substrate, encapsulated by GroEL:ES to adopt a structure virtually identical to that in the MetK tetramer (12). How the physico-chemical environment of the GroEL:ES chamber modulates the energy landscape of the folding reaction remains only partially understood (Fig. 1*A*). For several proteins, including model substrates and authentic client proteins, in-cage folding has been observed to be substantially accelerated (10 to 100-fold) compared to folding in bulk solution (in the absence of aggregation) (13–23). Entropic confinement of the encapsulated protein, lowering kinetic folding energy barriers (Fig. 1*A*), as well as interactions with flexible C-terminal Gly-Gly-Met repeat sequences protruding from the equatorial GroEL domains into the cage have been implicated in mediating accelerated folding (13–15, 20, 22–24). Consistent with experimental data (14, 22, 25), computational analyses of the thermodynamics of the aqueous solvent experienced by the encapsulated protein suggested that a repulsion between the hydrophilic (net negatively charged) cavity wall and hydrophobic parts of the nonnative client strengthens the hydrophobic effect, enforcing a more compact shape (26, 27).

Challenging this view, recent observations with a highly unstable dihydrofolate reductase (DHFR) construct from a cold-adapted bacterium suggested that the environment of the chaperonin cage favors unfolding (28) due to a diminished hydrophobic effect (29). Here, we investigated the potential mechanistic implications of these findings for understanding chaperonin function. To analyze the effect of encapsulation on thermodynamic protein stability, we generated a GroEL/ES-dependent variant of the mesophilic maltose-binding protein (MBP) by introducing four destabilizing mutations, henceforth named quadruple mutant-MBP (QM-MBP). Thermal unfolding experiments in combination with biochemical and biophysical analyses showed that the folded QM-MBP encapsulated in the GroEL:ES cage is markedly stabilized compared to the protein in free solution. We suggest that a stabilizing environment contributes to efficient in-cage folding of chaperonin-dependent proteins.

## Results

### QM-MBP, A Destabilized Variant of MBP.

Temperature-dependent unfolding, monitored spectroscopically, provides a direct measure of a protein’s thermodynamic stability (30). By comparing the melting temperature (*T*_m_) of a folded protein inside the GroEL:ES cage and in solution, we should therefore be able to assess the effect of the chaperonin environment on client protein stability. A functionally relevant model protein should satisfy the following criteria: i) Its *T*_m_ is substantially below that of GroEL:ES; ii) Folding rate and/or yield is enhanced by GroEL/ES compared to spontaneous folding, indicating that the protein interacts productively with the chaperonin system; and iii) Folding and unfolding is measurable in real time while the protein is encapsulated.

Taking advantage of the fact that GroEL and GroES lack tryptophan (Trp) residues (31), we chose *E. coli* MBP (41 kDa) as an established model protein. MBP is a discontinuous two-domain protein containing eight Trp residues (Fig. 1*B*; PDB: 1OMP). Folding was monitored by recording the robust increase in intrinsic Trp fluorescence as MBP converts from its unfolded to the folded state (14). While wild-type MBP (WT-MBP) folds efficiently without chaperonin assistance in vitro, substitutions V8G and Y283D in the N-domain (Fig. 1*B*), generating double mutant MBP (DM-MBP), have been shown to confer GroEL/ES dependence (14, 15, 17). However, the *T*_m_ of WT- and DM-MBP measured by nano differential scanning fluorimetry (nanoDSF) is above 60 °C (Fig. 1*C* and *SI Appendix*, Fig. S1*A*), too close to the reported stability of GroEL, GroES, or the GroEL:ES complex (>70 °C) (32, 33). Thus, we needed to generate a mutant version of MBP with a substantially lower *T*_m_. We noted that His64 in the N-domain of MBP forms long-distance backbone hydrogen-bond contacts to Met330 and Val261, while Arg316 in the C-domain forms a hydrogen bond with Tyr242 and is in electrostatic interaction with Asp236 (Fig. 1*B*). Removal of these interactions by nearly isosteric replacements was expected to weaken the interface between the N- and C-domains and lower the stability of MBP. We therefore introduced two additional mutations, H64F and R316Q, into DM-MBP, generating the quadruple-mutant QM-MBP (Fig. 1*B*). The cooperative unfolding transition of QM-MBP decreased to a *T*_m_ of 44.3 ± 0.11 °C, 18 °C lower than DM-MBP (61.8 ± 0.14 °C) and WT-MPB (63.6 ± 0.13 °C) (Fig. 1*C* and *SI Appendix*, Fig. S1*A*). Upon addition of the ligand maltose, which binds in a deep cleft between the N- and C-domains (*SI Appendix*, Fig. S1*B*), the *T*_m_ of QM-MBP increased to 51.3 ± 0.13 °C, a value still far lower than the *T*_m_ of maltose-bound WT- and DM-MBP (75.6 ± 0.04 and 74.8 ± 0.08 °C, respectively) (Fig. 1*C* and *SI Appendix*, Fig. S1*A*) and of GroEL:ES (71.6 ± 0.17 °C). This indicated that although QM-MBP is strongly destabilized, it is capable of folding into a maltose-binding, native-like conformation.

The spontaneous folding rate of QM-MBP at 20 °C was very slow with a t_1/2_ ~10 h (Fig. 1*D*), compared to WT- and DM-MBP with t_1/2_ values of ~25 s and ~30 min, respectively (14). Notably, the QM-MBP folding rate was independent of protein concentration in the range of 50 to 200 nM (Fig. 1*D*), indicating the absence of reversible aggregates that might slow folding (14). Consistent with this finding is the failure to detect any light scattering (turbidity) signal in refolding reactions even at a protein concentration of 500 nM (*SI Appendix*, Fig. S1*C*). In contrast, the aggregation-prone protein MetF, an obligate client protein of GroEL/ES, showed turbidity during attempted refolding already at 200 nM (21) (*SI Appendix*, Fig. S1*C*). GroEL/ES accelerated QM-MBP folding ~20-fold to a t_1/2_ of ~28 min (Fig. 1*E*), consistent with previous findings for DM-MBP and other proteins (13–15, 17, 18, 20, 21). When GroEL, GroES and ATP were added 30 or 60 min after diluting QM-MBP from denaturant into refolding buffer, the accelerated rate and full folding yield were maintained, arguing further against aggregation during spontaneous folding (Fig. 1*E* and *SI Appendix*, Fig. S1*C*).

In summary, the quadruple mutant of MPB, QM-MBP, is thermodynamically destabilized and requires kinetic assistance by GroEL/ES for efficient folding, similar to in vivo chaperonin clients such as Rubisco large subunit (RbcL), DapA, and MetF (13, 18, 21).

### QM-MBP Is Structurally Stabilized Inside the GroEL:ES Cage.

**Fig. 2.**
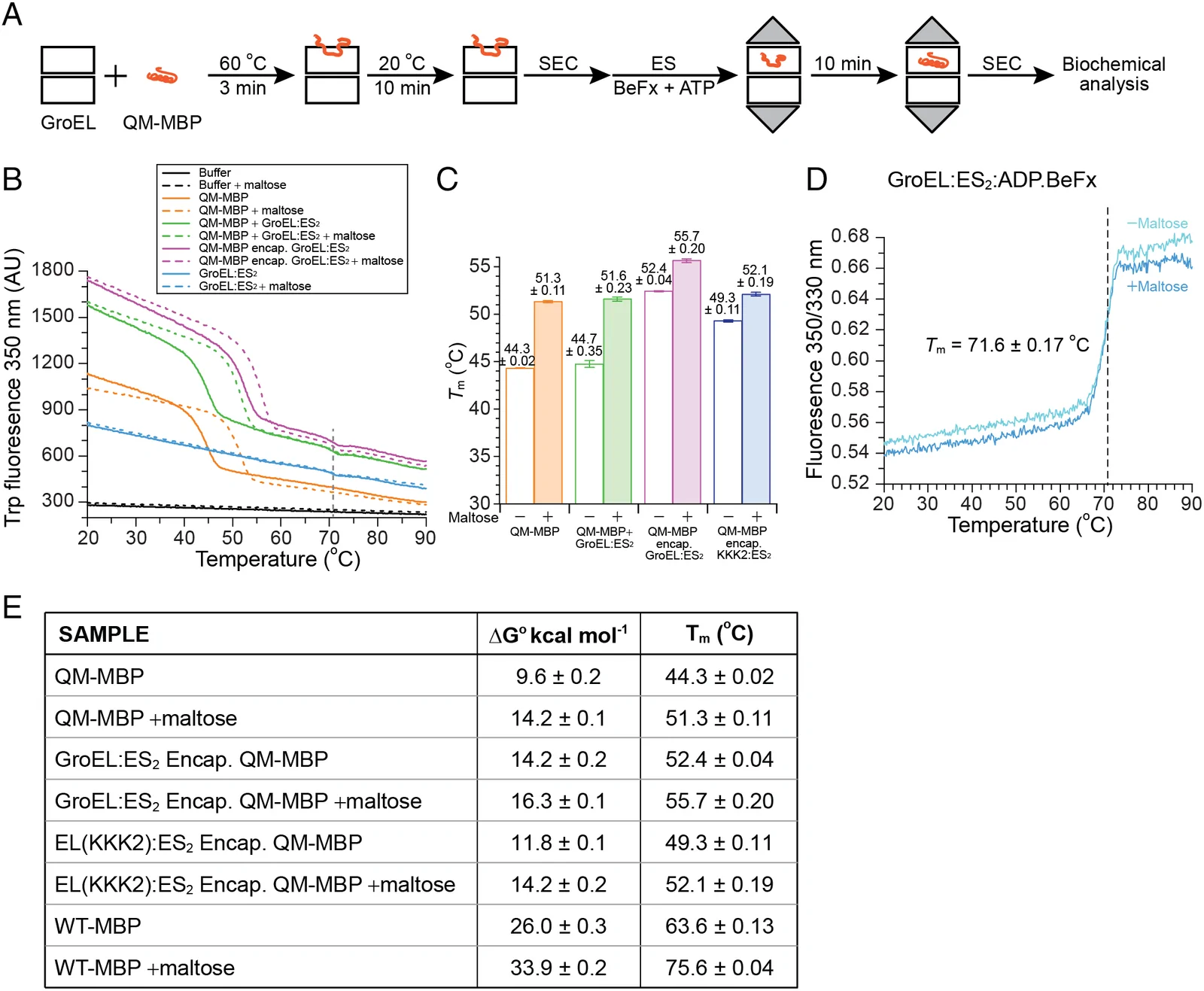
Stability of chaperonin-encapsulated QM-MBP. (*A*) Schematic depicting the preparation of GroEL:ES-encapsulated QM-MBP. To allow binding of unfolded QM-MBP to GroEL, the proteins were incubated at 60 °C for 3 min in refolding buffer A, followed by cooling at 20 °C for 10 min. The GroEL:QM-MBP complex was isolated by size-exclusion chromatography (SEC), and 5 μM complex mixed with GroES (20 μM), BeFx (1 mM), and ATP (200 μM) to induce encapsulation. The ternary complex was isolated by SEC. (*B*) Thermal denaturation of chaperonin-encapsulated QM-MBP (1 µM). Melting curves in presence or absence of 10 mM maltose in refolding buffer A containing 1 mM BeFx were recorded by differential scanning fluorimetry (nano-DSF) at 350 nm emission over a temperature range of 20 to 90 ^o^C using a heating rate of 5 °C min^−1^. For comparison, melting curves of free QM-MBP in presence or absence of chaperonin complex and chaperonin complex alone are also shown (n = 3). The *T*_m_ of the chaperonin complex is indicated [dashed vertical line, see (*D*)]. (*C*) Comparison of the thermal stability of free and chaperonin-encapsulated QM-MBP. *T*_m_ values obtained from melting curves in (*B*) are shown in bar representation (mean ± SEM, n = 3). (*D*) Thermal denaturation of the GroEL:ES_2_:ADP·BeFx complex. The thermal denaturation of the chaperonin complex was monitored using the ratio of fluorescence emission at 350 to 330 nm wavelength in presence or absence of maltose. The derived *T*_m_ value is indicated by a dashed line. Representative melting curves are shown (n = 3). (*E*) Thermodynamic stability of free and chaperonin-encapsulated MBP. ΔG°, Gibbs free energy of unfolding; Encap., encapsulated.

We next investigated the thermodynamic stability of QM-MBP when encapsulated in GroEL:ES. To allow highly efficient chaperonin–substrate complex formation, we briefly incubated QM-MBP and GroEL at 60 °C, which results in rapid QM-MBP unfolding and binding to GroEL (34) (Fig. 2*A*). Note that the GroEL tetradecamer is stable up to 73.5 ± 0.17 °C, as measured by circular dichroism (CD) (*SI Appendix*, Fig. S2*A*). The reaction was then cooled to 20 °C, followed by addition of GroES, BeFx (beryllium fluoride), and ATP, resulting in the formation of a noncycling GroEL:ES_2_:ADP^.^BeFx complex (35–38) with QM-MBP captured in the chaperonin cage (Fig. 2*A*). The chaperonin complex containing the trapped QM-MBP was then isolated by size exclusion chromatography (SEC) (Fig. 2*A* and *SI Appendix*, Fig. S2*B*). Using nanoDSF, we determined that the *T*_m_ of encapsulated QM-MBP was 52.4 ± 0.04 °C without maltose and 55.7 ± 0.20 °C with maltose (Fig. 2 *B* and *C*). These values are higher by ~8 °C and ~4 °C, respectively (Fig. 2 *B* and *C*), than those measured for QM-MBP in free solution (Fig. 1*D* and *SI Appendix*, Fig. S1*A*). As a control, the *T*_m_ of free QM-MBP, plus or minus maltose, remained unchanged when incubated in the presence of GroEL:ES_2_:ADP^.^BeFx complexes not containing encapsulated protein (Fig. 2 *B* and *C*). By monitoring the ratio of fluorescence intensity at 350 nm to 330 nm, we determined that the *T*_m_ of the GroEL:ES_2_:ADP^.^BeFx complex was 71.6 ± 0.17 °C (Fig. 2*D*), consistent with previous findings (32, 33) and the reported stability of GroEL alone (*SI Appendix*, Fig. S2*A*). Since the *T*_m_ values of the chaperonin complex and of QM-MBP are well separated (Fig. 1*D*), our findings indicate that the chaperonin cage has a stabilizing effect on the encapsulated, folded QM-MBP.

Next, to investigate whether the negative net charge of minus 42 of the inner wall of the GroEL:ES cage contributes to stabilizing the encapsulated QM-MBP, we used the GroEL mutant EL(KKK2), in which two cavity-exposed aspartates (Asp351; Asp361) and one glutamate residue (Glu363) per GroEL subunit are replaced with lysines, resulting in a net charge of the cavity wall of zero (14). Indeed, the EL(KKK2):ES cage had a reduced stabilizing effect on the encapsulated QM-MBP, resulting in a *T*_m_ of 49.3 ± 0.11 °C (+5 °C relative to bulk solution) compared to 52.4 ± 0.04 °C for GroEL:ES (+8 °C relative to bulk solution) (Fig. 2 *C* and *E* and *SI Appendix*, Fig. S2*C*). This is consistent with predictions from simulation that the charged wall character enhances protein compaction (26).

To estimate the extent of stabilization experienced by the encapsulated QM-MBP, we calculated the Gibbs free energy of unfolding (Δ*G*^o^) of QM-MBP and WT-MBP from the thermal denaturation curves (*Materials and Methods*). The Δ*G*^o^ values for QM-MBP unfolding in solution and when encapsulated in GroEL:ES were 9.6 ± 0.2 and 14.2 ± 0.2 kcal mol^−1^, respectively (Fig. 2*E*), reflecting a marked stabilization by 4.6 kcal mol^−1^ by the chaperonin cage. The Δ*G*^o^ value was 11.8 ± 0.1 kcal mol^−1^ for encapsulation in EL(KKK2):ES, corresponding to a ~50% reduced stabilization of 2.2 kcal mol^−1^ (Fig. 2*E*).

These data demonstrate that encapsulation of QM-MBP inside GroEL:ES results in a substantial stabilization against unfolding compared to the protein in solution, suggesting that a thermodynamically stabilizing effect on encapsulated protein contributes to efficient chaperonin-mediated folding.

### Analysis of QM-MBP Structural Compactness by smFRET.

**Fig. 3.**
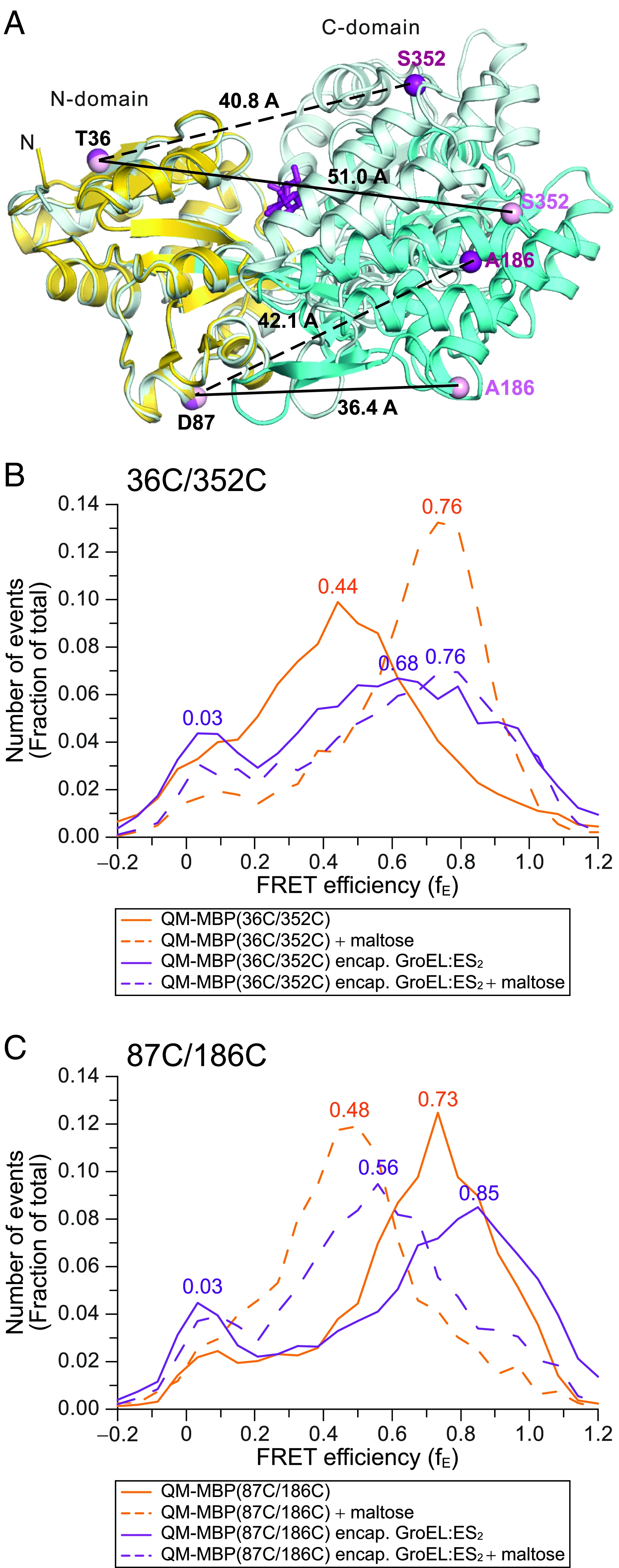
Probing the compaction of chaperonin-encapsulated QM-MBP by single molecule fluorescence resonance energy transfer (smFRET). (*A*) Location of FRET probes at residue pairs Thr36/Ser352 and Asp87/Ala186 in the crystal structures of maltose-bound (PDB: 1ANF) and ligand-free MBP (PDB: 1OMP; coloring as in Fig. 1*B*). The respective Cα positions are indicated by purple spheres. Distances are indicated in Å. Maltose bound to the closed state of MBP is shown as a stick model. Of note, the ligand-free conformation in the crystal lattice is probably partially compacted compared to the open state in solution. (*B* and *C*) FRET efficiency (f_E_) histograms for labeled QM-MBP(36C/352C) (*B*) and QM-MBP(87C/186C) (*C*). Chaperonin-encapsulated and free protein at 100 pM in presence or absence of 10 mM maltose in FRET buffer C was analyzed by smFRET. Distributions in presence of maltose are indicated by dashed lines.

Upon maltose binding, the N- and C-terminal domains of MBP, connected by a hinge, undergo a “Venus flytrap”-like closing motion around the ligand, an induced-fit mechanism involving residues from both domains (39–41). The N-domain of MBP behaves like a rigid body in this conformational transition, while the C-domain exhibits more plasticity (Fig. 3*A*). We also noted, based on the *T*_m_ analysis above, that the relative stabilization by maltose binding was lower for the encapsulated QM-MBP (from 52.4 to 55.7 °C) than for QM-MBP in solution (44.3 to 51.3 °C) (Fig. 2*E*), suggesting that confinement in chaperonin alone may already induce a partially closed conformation. To directly analyze the effect of the chaperonin environment on structural compactness of QM-MBP, we employed single molecule fluorescence resonance energy transfer (smFRET), where changes in FRET efficiency (f_E_) between donor and acceptor fluorophores reflect changes in residue distances (25, 42–44). To this end, we mutated either the residue pair Thr36/Ser352 or Asp87/Ala186 to cysteines (WT-MBP lacks cysteines) and labeled them with Alexa-555 (donor) and Alexa-647 (acceptor) fluorophores (*Materials and Methods*). The molecular distance for these residue pairs is sensitive to maltose binding, as shown for WT-MBP (40, 41) (Fig. 3*A*).

Positions 36 and 352 are located at the top of the N- and C-domains of MBP crystal structures (PDB: 1OMP; PDB: 1ANF), respectively, and maltose binding reduces their distance by ~10 Å (Fig. 3*A*). This is reflected in a shift in the distribution of f_E_ values from around 0.44 to 0.76 for QM-MBP in solution (Fig. 3*B*). The f_E_ distribution in the absence of maltose is wider, suggesting a broader distribution of states (Fig. 3*B*). Interestingly, upon encapsulation with GroES and folding inside GroEL:ES (without maltose), the major conformational distribution shifted to higher f_E_ values centering around f_E_ 0.68, compared to f_E_ 0.44 in solution (Fig. 3*B*), consistent with the chaperonin cavity favoring relatively more compact states. Addition of maltose resulted in some further compaction to f_E_ 0.76. This maltose-induced shift is smaller than for QM-MBP in solution (Fig. 3*B*), suggesting that encapsulation per se results in a compaction along the trajectory surveilled by the T36C/S352C FRET pair. This interpretation is consistent with the limited additional stabilization of the encapsulated protein by maltose binding (Fig. 2*E*). Notably, a minor conformational distribution around a low f_E_ of 0.03, not responsive to maltose, was also observed (Fig. 3*B*), presumably representing a small amount of misfolded or not successfully encapsulated QM-MBP. The latter possibility is consistent with QM-MBP populating mainly highly expanded states when bound to the GroEL apical domains (*SI Appendix*, Fig. S3*A*), similar to previous observations for DM-MBP (25). Moreover, the f_E_ distribution of GroEL-bound QM-MBP differed from the conformational ensemble of the kinetically trapped folding intermediate(s) populated during the slow spontaneous folding of QM-MBP (*SI Appendix*, Fig. S3*A*).

In contrast to the Thr36/Ser352 residue pair, the distance of residues Asp87 and Ala186, located on the opposite face of the MBP domains, increases by ~6 Å upon transition to the closed, maltose-bound conformation (39, 40) (Fig. 3*A*). Indeed, the f_E_ distribution of QM-MBP in solution shifted from f_E_ 0.73 to f_E_ 0.48 (Fig. 3*C*). Again, the conformational distribution of the encapsulated QM-MBP in the absence of maltose (f_E_ 0.85) was more compact along the Asp87-Ala186 trajectory than for the protein in solution (f_E_ 0.73) (Fig. 3*C*). Notably, a clear shift to lower f_E_ values occurred upon maltose addition, although the f_E_ distribution of the encapsulated protein (f_E_ 0.56) remained more compact than that of the free protein (f_E_ 0.48) (Fig. 3*C*). A broad, bimodal distribution was recorded for the GroEL-bound, unfolded protein with highly expanded (f_E_ ~0.06) and compact (f_E_ ~0.9) states along the Asp87-Ala186 trajectory (*SI Appendix*, Fig. S3*B*). The kinetically trapped intermediates formed during spontaneous folding displayed a broad distribution of states, including conformations with higher f_E_ values along the 87C-186C trajectory than in native QM-MBP in solution (*SI Appendix*, Fig. S3*B*), suggesting the presence of nonnative interactions.

In summary, encapsulation in the GroEL:ES complex induces a compacted state of QM-MBP relative to the protein in solution along the distance trajectories analyzed, both in the absence and presence of maltose. This is consistent with the thermodynamic stabilization afforded by the chaperonin cage.

### Conformational Stability of Chaperonin-Encapsulated QM-MBP at Peptide Resolution.

**Fig. 4.**
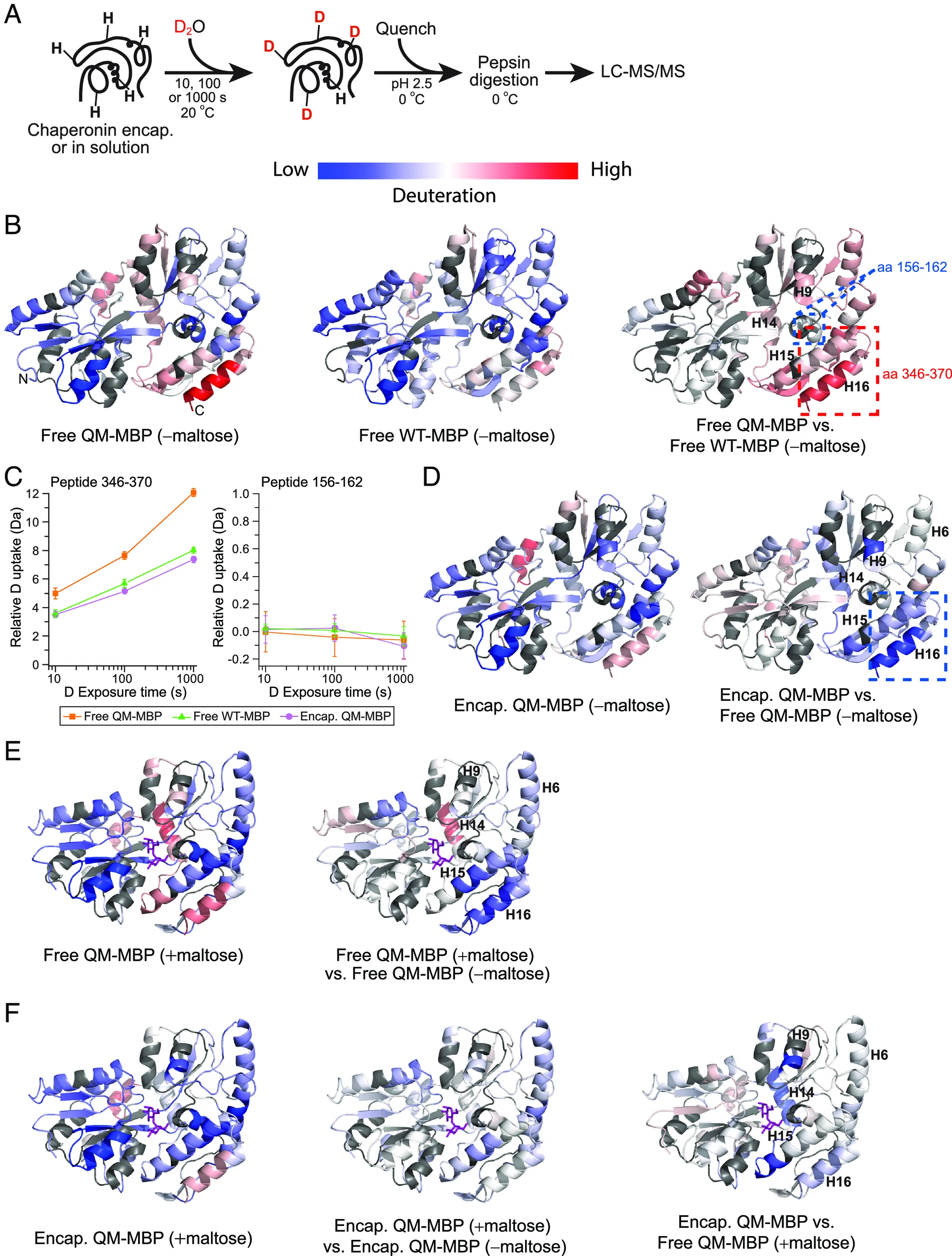
Structural analysis of chaperonin-encapsulated QM-MBP by hydrogen/deuterium exchange (H/DX). (*A*) Schematic of the H/DX workflow in deuterated refolding buffer A. (*B*) Deuterium incorporation into free QM-MPB and WT-MBP (1 µM final concentration), and their differential H/DX protection in the absence of maltose. Peptide-resolution H/DX data at 1,000 s incorporation time for 27 peptides (*SI Appendix*, Fig. S4) were mapped onto the MBP crystal structure (PDB: 1OMP). The blue–white–red color gradient represents 0 to 50% incorporation of deuterium into backbone amides for the respective proteins and −25% to +25% for the comparison. Blue regions indicate protection from exchange (stabilization), while red regions indicate deprotection (destabilization). Regions of the MPB sequence with no H/DX information are in dark gray. N- and C-termini, as well as helices H15 and H16 in the C-domain and peptides 156 to 162 and 346 to 370 are highlighted. (*C*) Time-resolved deuterium incorporation for representative peptides near the substrate-binding pocket (residues 156 to 162) and in the C-domain (residues 346 to 370) of MBP proteins in the absence of maltose. Data are shown for WT-MBP (green), free QM-MBP (orange), and chaperonin-encapsulated (Encap.) QM-MBP (purple). Error bars represent SD (n = 3). (*D–F*) Deuterium incorporation into chaperonin-encapsulated and free QM-MBP in the absence or presence of maltose (10 mM), as indicated. Chaperonin-encapsulated QM-MBP (minus maltose) and its comparison to free QM-MBP (minus maltose), corresponding to 33 overlapping peptides (*SI Appendix*, Fig. S4) mapped onto the MBP crystal structure (PDB: 1OMP) (*D*); free QM-MBP in the presence of maltose and its comparison to free QM-MBP (minus maltose) mapped onto the MBP crystal structure (PDB: 1ANF) (*E*); chaperonin-encapsulated QM-MBP with maltose and its comparison to encapsulated QM-MBP (minus maltose) or its comparison to free QM-MBP (plus maltose) (*F*). Color coding as in (*B*).

We next used hydrogen–deuterium exchange (H/DX) coupled to mass spectrometry (LC–MS/MS) to obtain more insight into the structural dynamics of chaperonin-encapsulated QM-MBP based on the principle that stable secondary structure is associated with H/DX protection of backbone amides (45). Note that previous analyses with the obligate GroEL substrate DapA did not detect any nonspecific effects of encapsulation on H/DX properties (18). We incubated encapsulated QM-MBP or free QM-MBP with a deuterated buffer at 20 °C for different times (0, 10, 100, and 1,000 s) followed by acid-quenching of exchange at 0 °C (Fig. 4*A*). To obtain information at peptide resolution, the proteins were then subjected to pepsin digestion and LC–MS/MS (Fig. 4*A*). We identified 33 peptides in both samples, covering ~80% of the QM-MBP sequence (*SI Appendix*, Fig. S4). For comparison, free WT-MBP was also analyzed, with 27 peptides corresponding to those of QM-MBP (*SI Appendix*, Fig. S4).

Folded QM-MBP in solution was substantially destabilized in the C-domain relative to WT-MBP, consistent with mutations in QM-MBP weakening the interface between the N- and C-domains. This destabilizing effect was most notable in residues 110 to 148, 168 to 214, and 311 to 370, which include the C-terminal helices H14-H16 (Fig. 4 *B* and *C* and *SI Appendix*, Figs. S4 and S5). In contrast, large parts of the N-domain including residues 23 to 61 and 76 to 95, as well as parts of the maltose binding pocket (peptides 156 to 162 and 248 to 262) showed high exchange protection, similar to WT-MBP (Fig. 4 *B* and *C* and *SI Appendix*, Figs. S4 and S5). Encapsulation of QM-MBP in GroEL:ES resulted in an overall increase in H/DX protection of the C-domain (Fig. 4*D*), Specifically, deuterium uptake in peptides 210 to 214 (H9), 311 to 322 (H14), 340 to 359, and 346 to 370 (H15 and H16), destabilized in free QM-MBP, was markedly reduced in the encapsulated protein (Fig. 4 *B* and *D* and *SI Appendix*, Fig. S5), offsetting the mutation-induced dynamics. In fact, peptide segments 210 to 214, 340 to 359, and 346 to 370 reached a degree of protection upon encapsulation similar to or exceeding that of free WT-MBP (Fig. 4 *B*–*D* and *SI Appendix*, Fig. S5). On the other hand, the protected peptide156 to 162 showed similar exchange properties in both the encapsulated and free proteins (Fig. 4*C* and *SI Appendix*, Fig. S5), arguing against nonspecific effects of encapsulation.

Maltose binding stabilized free QM-MBP mainly in the C-domain (H6, H15, H16) (Fig. 4*E*), consistent with the thermal stabilization afforded by maltose (*T*_m_ shift from 44.3 to 51.3 °C) (Fig. 2*E*). Interestingly, the stabilizing effect of maltose was remarkably similar to that of encapsulation (without maltose), strongly affecting helices H15 and H16 (Fig. 4*E*). This explains the more modest increase in melting temperature (*T*_m_ shift from 51.3 to 55.7 °C) upon maltose binding to the encapsulated protein (Fig. 2*E*), supporting the notion that encapsulation favors a state similar to the maltose-bound conformation (Fig. 4*F*). A notable exception is H14 (residues 315 to 327), which was destabilized upon maltose binding to free QM-MBP (Fig. 4*E*) but was stabilized when the protein was encapsulated (Fig. 4*F*).

Together these findings further support the conclusion from the thermal unfolding and smFRET experiments that the environment of the GroEL:ES chamber structurally stabilizes the folded state of encapsulated QM-MBP.

### Reversibility of QM-MBP Unfolding in the GroEL:ES Cage.

GroEL/ES-assisted folding typically involves multiple interaction cycles in which not-yet folded protein first interacts with the hydrophobic binding regions of the GroEL apical domains, followed by GroES-mediated encapsulation for in-cage folding (3). To characterize the properties of the chaperonin cage, it was of interest whether unfolding and refolding is possible while the client protein remains encapsulated, thereby circumventing apical domain binding. To address this question, we first tested if GroES remains stably bound to GroEL upon incubation at increasing temperatures. Exchange of bound GroES with excess GroES carrying an Alexa-555 fluorophore (ES-555) at an engineered C-terminal cysteine (ES98C) was monitored by size exclusion chromatography, separating GroEL-bound from free GroES (*SI Appendix*, Fig. S6*A*). Reactions maintained at 20 °C showed essentially no exchange, and exchange was only minor after incubation at 40 °C (*SI Appendix*, Fig. S6 *B* and *C*). However, clear exchange of bound GroES occurred upon heating to 55 °C (*SI Appendix*, Fig. S6*D*). Notably, this effect was accompanied by the loss of encapsulated QM-MBP from the chaperonin complex, consistent with opening of GroEL:ES and protein release (*SI Appendix*, Fig. S*6D*). Detailed analysis of the temperature dependence of this phenomenon revealed that GroES exchange was half-maximal at ~50 °C, close to the *T*_m_ of encapsulated QM-MBP (Fig. 2*C*), reaching a maximum at 55 °C (*SI Appendix,* Fig. S6*E*). Surprisingly, we found that the more stable DM-MBP, which has a *T*_m_ of 61.8 °C in solution (Fig. 1*C*), i.e., higher than the temperature of GroES exchange, was retained inside the chaperonin at 55 °C (*SI Appendix*, Fig. S6*F*). Thus, exit of QM-MBP, under these conditions, apparently required unfolding, which did not occur for DM-MBP. This suggested that the cage remains “operationally” closed at 55 °C, with GroES dissociation either being transient or occurring at the level of individual subunits, retaining folded protein but allowing unfolded protein to escape, perhaps through fenestrations between GroEL and GroES.

**Fig. 5.**
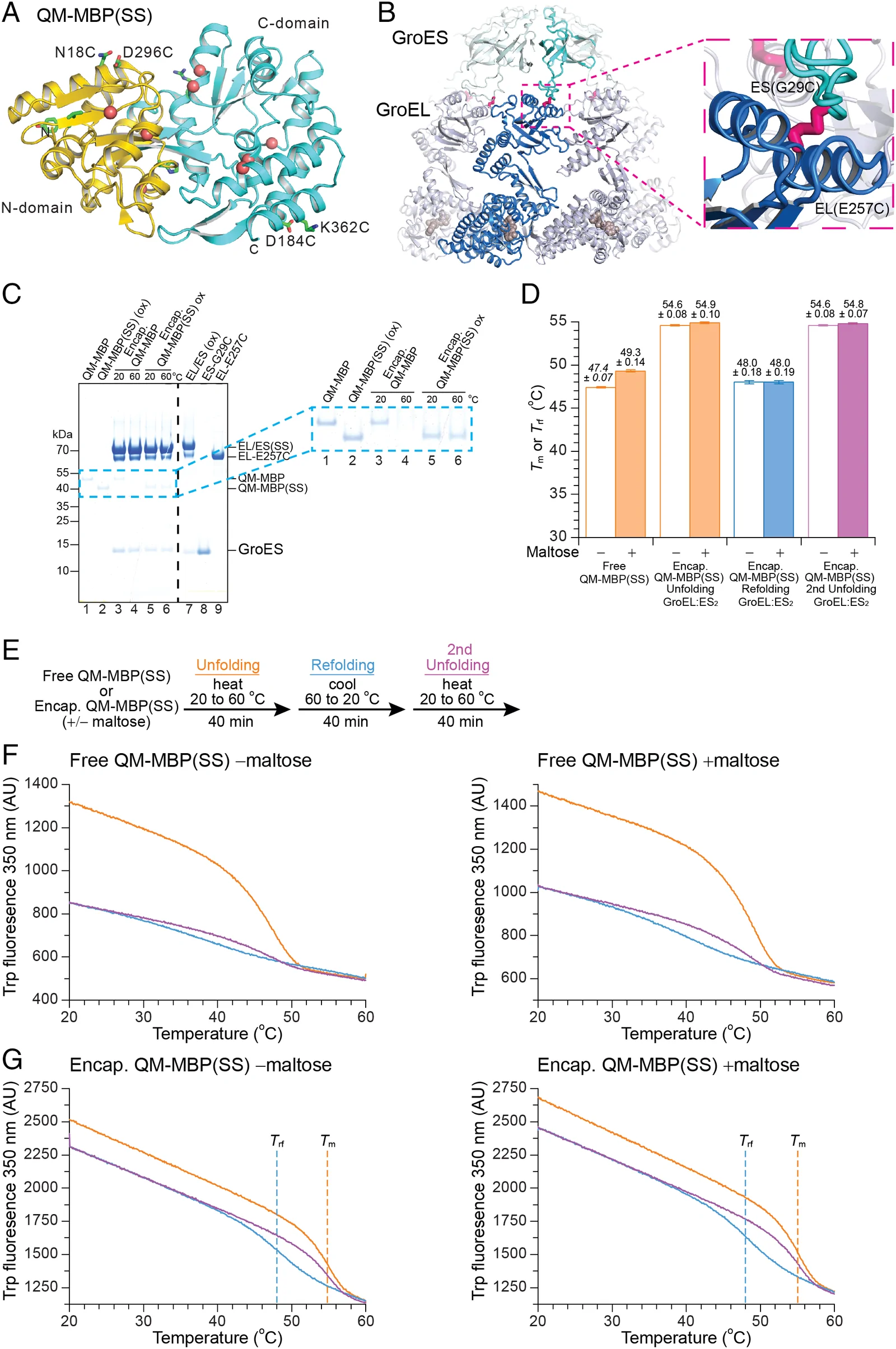
Reversible thermal unfolding and refolding of QM-MBP(SS) inside a disulfide-locked GroEL:ES cage. (*A*) Residues substituted in QM-MBP to generate QM-MBP(SS) are shown in stick representation. The cysteine substitutions in the N- and C-domains, as well as the residue substitutions in QM-MBP are shown in stick representation. Tryptophan (Trp) residues are shown as spheres (salmon). N- and C-termini are indicated. (*B*) AlphaFold 3 model for the EL(E257C):ES(G29C) chaperonin cage. GroEL and GroES subunits are shown in ribbon representation in bluewhite and pale cyan, respectively, with one pair of the subunits highlighted in marine and cyan, respectively. The intermolecular disulfide bridge between E257C and G29C is shown in magenta (magnified on the right). GroEL-bound ADP-Mg is shown in space-filling mode. (*C*) SDS-PAGE analysis of disulfide-bonded GroEL:ES with encapsulated substrate before and after heating. GroEL:ES(SS)-encapsulated QM-MBP or QM-MBP(SS) (2 µM complex) was incubated at 60 °C for 3 min in refolding buffer, then cooled to 20 °C for 10 min, followed by SEC separation and analysis by SDS-PAGE and Coomassie-blue staining. Control reactions were incubated at 20 °C. Insert shows a magnified, higher contrast image of the QM-MBP/QM-MBP(SS) bands (lanes 1 to 6). (*D*) Comparison of the thermal stability of free and chaperonin-encapsulated QM-MBP(SS). *T*_m_ values obtained from unfolding and *T*_rf_ values from refolding curves in the presence or absence of maltose (10 mM) are shown in bar representation (mean ± SEM, n = 3). (*E*) Schematics for the reversible refolding of free or GroEL:ES(SS)-encapsulated QM-MBP(SS) in the presence or absence of 10 mM maltose. Thermal cycling was performed in three steps: unfolding (orange) from 20 to 60 °C, refolding (blue) from 60 to 20 °C and repeat unfolding (purple) from 20 to 60 °C; heating and cooling at 1 °C min^−1^. Unfolding and refolding curves were recorded using nanoDSF at 350 nm emission at 1 µM QM-MBP(SS). (*F* and *G*) Representative unfolding and refolding curves of free (*F*) and chaperonin-encapsulated QM-MBP(SS) (*G*) in presence or absence of maltose (n = 3). Vertical dashed lines indicate *T*_m_ (orange) and *T*_rf_ (blue).

Based on these considerations, we hypothesized that the presence of intramolecular disulfide bonds in QM-MBP may suffice to hinder its escape during in-cage unfolding. We introduced two disulfide bonds previously characterized in DM-MBP (15), N18C-D296C in the N-domain and D184C-K362C in the C-domain, to generate QM-MBP(SS) (Fig. 5*A*). In addition, to prevent GroES dissociation, we engineered cysteine mutants of GroEL and GroES, EL(E257C), and ES(G29C) respectively, to allow formation of a disulfide bond between the GroES mobile loop region and the apical GroEL domain (Fig. 5*B*). These mutants were designed using the program “Disulfide by Design” [v2.13; (46) and the GroEL:(ES)_2_ structure (PDB: 8P4M)]. Air oxidation of the mutant GroEL:(ES)_2_ complex in the presence of ATP^.^BeFx resulted in the majority of EL(E257C) to form the predicted disulfide bond with ES(G29C), as visualized by nonreducing SDS-PAGE (Fig. 5*C*, lane 7). Importantly, oxidized QM-MBP(SS), migrating faster than QM-MBP (Fig. 5*C*, lanes 1 and 2), was efficiently retained in the chaperonin cage at temperatures up to at least 60 °C (Fig. 5*C*, lanes 4 and 6). DSF analysis showed that oxidized, free QM-MBP(SS) was only mildly stabilized relative to QM-MBP (*T*_m_ 47.4 °C compared to 44.3 °C), with maltose having only little effect on the *T*_m_ (Fig. 5*D*), suggesting that the closed conformation is favored in the presence of the disulfide bonds. Notably, encapsulation in EL(E257C):ES(G29C) resulted in a marked stabilization of QM-MBP(SS), shifting the *T*_m_ of cooperative unfolding to 54.8 °C, close to the *T*_m_ of encapsulated QM-MBP in the presence of maltose (*T*_m_ 55.8 °C) (Figs. 5*D* and 2*C*). This result confirmed the stabilizing effect of the GroEL:ES cage under conditions where QM-MBP remained encapsulated during unfolding. Addition of maltose did not afford further stabilization of encapsulated QM-MBP(SS) (Fig. 5*D*), consistent with confinement inside GroEL:ES already favoring a conformationally more compact state.

Having established that QM-MBP(SS) is retained in the GroEL:ES complex during thermal unfolding, we next analyzed the reversibility of in-cage unfolding by DSF during temperature upshift from 20 to 60 °C and cooling to 20 °C, followed by a second unfolding cycle (Fig. 5*E*). As expected, free QM-MBP(SS) showed no discernible refolding transition after unfolding (Fig. 5*F*), irrespective of the presence of maltose, presumably due to heat-induced aggregation. Accordingly, there was only a very weak unfolding transition during the second unfolding attempt (Fig. 5*F*). In contrast, the chaperonin-encapsulated QM-MBP(SS) refolded efficiently after thermal unfolding (Fig. 5*G*). Interestingly, the transition temperature of refolding, *T*_rf_, of 48.0 ^o^C was lower than the *T*_m_ of unfolding (54.6 °C) (Fig. 5 *D* and *G*). A similar hysteresis effect was previously observed at intermediate concentrations of guanidinium hydrochloride (GuHCl) for unfolding and refolding of free DM-MBP, but not WT-MBP, and was attributed to the population of a kinetically trapped folding intermediate by the mutant MBP (15). A repeat unfolding cycle of the in-cage refolded QM-MBP(SS) reproduced the *T*_m_ of 54.6 ^o^C (Fig. 5 *D* and *G*), indicating that the protein had refolded to a native-like state in the chaperonin cage.

In summary, the chaperonin cage provides an environment that is conducive to folding. The in-cage folding reaction can be mechanistically uncoupled from the initial hydrophobic interactions of the nonnative client protein with the apical GroEL domains.

## Discussion

A salient feature of the GroEL/ES chaperonin mechanism is the transient encapsulation of unfolded client protein in the folding cage formed by the GroEL ring and the lid-shaped GroES. How exactly the physico-chemical environment of the chaperonin promotes folding remains incompletely understood. Does it merely passively prevent protein aggregation or actively modulate the folding energy landscape, and can in-cage folding be mechanistically uncoupled from initial binding of the nonnative protein to the apical GroEL domains? Here we have addressed some of these questions by modifying the GroEL/ES system to allow reversible in-cage unfolding of a conformationally destabilized model protein, QM-MBP. We demonstrate that the GroEL:ES complex provides a specialized environment that can accelerate protein folding, modulating the folding energy landscape, but also markedly stabilizes the folded end-state compared to the protein in bulk solution. This latter effect likely contributes to the efficiency of the chaperonin mechanism, especially when folded protein subunits reach their final stable state only upon downstream oligomeric assembly.

Compared to WT-MBP with a *T*_m_ of 63.6 ± 0.13 °C, QM-MBP contains four mutations that shift its *T*_m_ to 44.3 ± 0.11 °C, corresponding to a destabilization by 16.4 kcal/mol. Spontaneous folding of QM-MBP is ~1,000-fold slower than WT-MBP folding (14). A similar, though less pronounced, effect in kinetically disabling the folding pathway was previously observed for DM-MBP (sharing two of the mutations in QM-MBP) and was attributed to the population of kinetically trapped folding intermediates (15, 23). The chaperonin system partially rescues the kinetic folding defect by accelerating the folding rate of QM-MBP ~20-fold [~13-fold for DM-MBP (15)]. This acceleration is not due to prevention of aggregation but to an effect of confinement that reduces the ensemble of conformational states, thereby lowering the entropic cost of folding (15, 23, 47).

A key finding of this work is that the QM-MBP folded inside the GroEL:ES cage experiences a marked thermodynamic stabilization. NanoDSF analysis revealed an increase in the *T*_m_ of the encapsulated protein compared to the protein in bulk solution by 8.1 °C, corresponding to a stabilization by 4.6 kcal/mol. This effect is reflected in reduced hydrogen–deuterium exchange mainly in the C-domain of QM-MBP, which is destabilized by the mutations. The extent of thermodynamic stabilization was reduced by ~50% when QM-MBP was encapsulated in a mutant chaperonin lacking the negative net charge of the cavity wall. This is consistent with experimental data that cavity wall charges contribute critically to folding rate acceleration (14, 25). Computational analyses to explain the possible mechanism suggested a repulsion between the negative net charge of the cage wall and hydrophobic parts of the nonnative client protein, conferred by the properties of the confined aqueous solvent, that would strengthen the hydrophobic effect and enforce a conformational compaction (26, 27). Indeed, as demonstrated by smFRET, the chaperonin cavity shifted the enclosed QM-MBP into a more closed state resembling the conformation otherwise induced by maltose binding. This is consistent with a strong collapse-enhancing effect of the chaperonin cavity for DM-MBP measured using optical tweezers (22).

Our results are, seemingly, at odds with previous findings that the chaperonin cavity is thermodynamically destabilizing, diminishing the hydrophobic effect that would drive protein folding (28, 29). This conclusion was based on observations with the cold-adapted dihydrofolate reductase (DHFR) protein from the psychrophilic, pressure resistant bacterium *Moritella profunda* (*Mp*), fused to eGFP (28). The *T*_m_ value of *Mp*DHFR is 39 to 41 °C (28, 48), similar to QM-MBP, but fusion with eGFP massively destabilizes the protein to a *T*_m_ of 23 °C (28). Thus, at room temperature, ~50% of the molecules populate nonnative states. Further thermodynamic destabilization upon encapsulation was deduced indirectly by measuring the lag time between addition of enzyme substrate and the appearance of enzyme activity (28). Notably, the human DHFR homolog has been shown to fold efficiently upon encapsulation in GroEL:ES, with the temporal development of H/DX protection being similar to folding in free solution (49), arguing against destabilization by chaperonin. We suggest that the observations with the cold-adapted model protein may be explained with the bimodal physical character of the chaperonin cage. While the wall of the cavity exposes multiple conserved negatively charged residues, the cage also contains at its bottom a meshwork of unstructured, hydrophobic Gly-Gly-Met repeat sequences (so-called C-terminal tails) emanating from the equatorial GroEL domains (3). These GGM sequences are in contact with the encapsulated protein during folding and have been shown to contribute to accelerated folding (14, 15, 20, 22, 24), presumably by “fluidizing” kinetically trapped folding intermediates through transient hydrophobic interactions. Given the limited intrinsic driving force of *Mp*DHFR (when fused to eGFP) to fold at normal temperature, it seems plausible that interactions with these sequences shift the folding equilibrium further toward the unfolded state, perhaps similar to the tethering of a SH3 protein folding intermediate inside GroEL observed by NMR (47). In contrast, in the case of the foldable QM-MBP, the GGM repeats may help to lower energy barriers between kinetic folding intermediates, as shown for DM-MBP (15, 44).

In-cage folding is normally preceded by binding of the nonnative protein to the apical GroEL domains from which it is displaced during the encapsulation process. This step may serve to reverse misfolded states (3, 5, 8), preparing the protein for productive in-cage folding. It was therefore of interest whether the closed cage allows reversible unfolding, circumventing this binding step. Surprisingly, we found that QM-MBP escaped from GroEL:ES upon thermal unfolding, whereas the more stable DM-MBP remained entrapped. This suggested that the unfolded protein may exit through gaps between GroEL and GroES that are too small for a folded protein to pass, consistent with a recent cryo-EM structure showing that an extended nascent polypeptide may thread through the space between GroEL and GroES (50). Indeed, introducing two disulfide bonds into QM-MBP and additionally disulfide-linking GroES to GroEL prevented the escape of QM-MBP(SS) upon unfolding. In-cage QM-MBP(SS) unfolding was fully reversible, with a second unfolding cycle reproducing the *T*_m_ of the first unfolding reaction. Thus, in-cage folding can be mechanistically uncoupled from protein binding to the apical GroEL domains.

Taken together, our results support an active cage-model of chaperonin-assisted folding in which the environment of the GroEL:ES cage promotes client protein compaction and formation of a thermodynamically stabilized native-like state.

## Materials and Methods

### Strains and Plasmids.

The *E. coli* DH5α strain was used for cloning purposes, and *E. coli* BL21(DE3) Gold (Stratagene) was utilized for protein expression.

Plasmids of QM-MBP (quadruple-mutant MBP, containing mutations V8G, H64F, Y283D, R316Q) and its cysteine variants, QM-MBP-NC (N18C, D184C, D296C, K362C), QM-MBP (T36C, S352C), and QM-MBP (D87C, A186C) were constructed by modifying the previously published pCH-MBP5(V8G/Y283D) plasmid (17) using the Q5 site-directed mutagenesis kit (NEB). Note that the MBP plasmids do not contain the signal sequence that would direct the proteins into the periplasm. GroEL(E257C), GroES(G29C), and GroES(98C) were likewise generated from the plasmids pET11a-GroEL and pET11a-GroES, respectively (38).

### Protein Expression and Purification.

Wild-type GroEL, GroES, and their cysteine mutants (51, 52) as well as WT-MBP and DM-MBP (14, 25), were expressed and purified as previously described. QM-MBP and QM-MBP(SS) were expressed in BL21 (DE3) cells. Cells were grown at 37 °C with shaking at 130 rpm for 2.5 to 3 h until OD_600_ of ~0.6, followed by cooling to 18 °C before inducing expression with 0.4 mM isopropyl-β-D-thiogalactopyranosid (IPTG) overnight. Cells from a 1 L culture were harvested by centrifugation (Beckman J6-MI centrifuge with a JS-4.2 rotor, 5000×*g*, 45 min at 4 °C). The cell pellet was resuspended in 50 mL lysis buffer [50 mM Tris-HCl, pH 7.5, 100 mM NaCl, 1 mg mL^−1^ lysozyme, 2.5 U mL^−1^ SmDNAse and Complete™, EDTA-free Protease Inhibitor Cocktail (Roche)] and sonicated with a tip sonicator (Misonix Sonicator 3000) on ice with the following settings: power output 7.5, pulse mode, 15 s on and 75 s pause, total time 5 min. The 50 mL suspension was aliquoted into 1.5 mL Eppendorf tubes, each tube containing 1 mL and centrifuged (Eppendorf 5427 R) at 15300×*g* for 30 min at 4 °C. QM-MBP and QM-MBP(SS) were purified from inclusion bodies (pellet) to eliminate contamination from host WT-MBP. Inclusion bodies were denatured in 200 µL 6 M GuHCl or 6 M GuHCl containing 10 mM DTT [QM-MBP(SS)] for 1 h at 20 °C (200 µM protein), followed by 200-fold dilution into refolding buffer A [20 mM MOPS-KOH pH 7.5, 100 mM KCl, 10 mM Mg(OAc)_2_] containing 1 µM GroEL, 2 µM GroES and 5 mM ATP. Chaperonin-assisted refolding was allowed to proceed for 3 h at 20 °C. Native QM-MBP or QM-MBP(SS) were subsequently purified on an amylose column (NEB) eluted with refolding buffer A containing 10 mM maltose, followed by size exclusion chromatography (SEC) on a Superdex-200 16/60 column (GE Healthcare), equilibrated in refolding buffer A. The purity was analyzed by SDS-PAGE and protein concentrations in the final preparations were determined by absorbance at 280 nm measured by a Nanodrop 1000 spectrophotometer (Thermo Fisher Scientific) using absorbance coefficients calculated based on the protein sequence with ProtParam (Swiss Institute of Bioinformatics), unless stated otherwise. Purified proteins were concentrated by ultrafiltration and snap-frozen in liquid N_2_ for storage at −80 °C or −70 °C.

### Preparation of GroEL-Bound and Chaperonin-Encapsulated QM-MBP.

To prepare GroEL-bound QM-MBP, 5 µM GroEL, and 10 µM QM-MBP were incubated at 60 ^o^C for 3 min in refolding buffer A to denature the QM-MBP, then cooled to 20 °C for 10 min. The reaction mixture (50 µL) was centrifuged (Eppendorf 5427 R at 15,300×*g* for 10 min) and then subjected to [Superose 6 Increase 3.2/300 column (Cytiva) equilibrated with refolding buffer A] to separate the GroEL-bound QM-MBP from free QM-MBP. For the production of chaperonin-encapsulated QM-MBP, the procedure followed the above binding protocol, with the exception that after the 10 min cooling step, the reaction was supplemented with 20 µM GroES, 200 µM ATP, and 1 mM BeFx (1 mM BeSO_4_, 10 mM NaF). The ternary complex was then isolated by SEC (Superose 6 Increase 3.2/300 column equilibrated with refolding buffer A containing 1 mM BeFx). The GroEL-bound or encapsulated fractions were pooled and concentrated. The concentration of QM-MBP (bound or encapsulated) was determined by densitometric comparison to free QM-MBP standards on SDS-PAGE. The complexes were kept on ice before use.

### Protein Labeling.

QM-MBP(T36C, S352C) and QM-MBP(D87C, A186C) were double-labeled with Alexa-555 and Alexa-647 (Invitrogen). Purified MBP (40 µM) was reduced in labeling buffer B [20 mM Tris-HCl pH 7.5, 100 mM KCl, 5 mM Mg(OAC)_2_] containing 1 mM TCEP for 15 min at room temperature (RT), and TCEP removed using a Nap-5 column (Cytiva) equilibrated with labeling buffer B. The reduced protein (10 µM) was incubated with a 10-fold molar excess of a 1:1 mixture of Alexa-555 and Alexa-647 dyes (100 µM) at 4 °C overnight. Labeled proteins were buffer exchanged using Nap-5 columns equilibrated with FRET buffer C [20 mM Tris-HCl pH 7.5, 20 mM KCl, 5 mM Mg(OAc)_2_, 10 mM DTT]. Labeling efficiency and protein concentrations were determined by Nanodrop-1000 spectrophotometry (Thermo Fischer Scientific). GroES(98C) was labeled with Alexa-555 using the same protocol as described for QM-MBP.

### smFRET.

The Alexa-555 and Alexa-647 double-labeled QM-MBP(T36C, S352C) and QM-MBP(D87C, A186C) proteins were used for smFRET. Analysis of the native, misfolded, GroEL-bound and chaperonin-encapsulated proteins was performed in presence or absence of 10 mM maltose. GroEL-bound and chaperonin-encapsulated fluorescent-labeled QM-MBP were prepared as above and isolated by SEC [Superose 6 Increase 3.2/300 column (Cytiva) equilibrated with FRET buffer C, without or with 10 mM BeFx]. The kinetically trapped state populated during spontaneous folding was prepared by 200-fold dilution of the denatured (6 M GuHCl, 10 mM DTT, at RT for 1 h), double-labeled QM-MBP into the FRET buffer for spontaneous refolding for 3 h before data acquisition (final protein concentration 100 pM). In the case of GroEL-bound or GroEL-encapsulated sample, the concentration of GroEL and GroES was 2 µM and 8 µM, respectively. For measurements in the presence of maltose, 10 mM maltose was included in the FRET buffer C. Fluorescence data were recorded for 15 min on a MicroTime 200 inverse time-resolved fluorescence microscope (PicoQuant) with pulsed interleaved excitation (PIE), as previously described (17, 43, 53). Two diode lasers at 640 nm (LDH-PC-640B) and at 530 nm (LDH-P-FA-530) were used. Each laser was set to 40 µW measured before the major dichroic mirror. Data were analyzed via the SymPhoTime software (PicoQuant) using a burst intensity method (54). A threshold of 25 photons per 1 ms bin was used to define an event. Three replicates were performed for each state and at least 500 events were analyzed in each replicate. The event distributions were similar among replicates. The events were pooled and the FRET efficiency histogram analyzed by normalizing to total events.

### Hydrogen/Deuterium Exchange Coupled to Mass Spectrometry (H/DX-MS).

H/DX-MS experiments were performed as previously described with minor modifications (55). WT-MBP, QM-MBP, and chaperonin-encapsulated QM-MBP were diluted 10-fold into deuterated refolding buffer A containing 1 mM BeFx to a final concentration of 1 µM, and incubated at 20 °C for varying times (0, 10, 100, 1,000 s). The exchange reactions were stopped by adding an equal volume of ice-cold quenching buffer D (100 mM sodium phosphate pH 2.1, 10 mM TCEP). Samples were injected into a Waters ACQUITY UPLC M-class liquid chromatography system, followed by on-line digestion via an Enzymate BEH-pepsin column (Waters) at a flow rate of 100 µL/min. Mass analysis was performed on a Waters Synapt G2Si mass spectrometer (Waters) using T-wave ion mobility as orthogonal peptide separation, and peptides were identified by analysis of MS^E^ data for five undeuterated control experiments using PLGS software (Waters). The peptide list was then imported into DynamX software (Waters), where the peaks from the spectra were manually checked and subsequently processed. Deuteration data for WT- and QM-MBP were mapped to the crystal structure of MBP (PDB:1OMP or 1ANF) by PyMOL (The PyMOL Molecular Graphics System, Version 3.0 Schrödinger, LLC). The color of amino acid residues was determined by the “B-factor” value generated by DynamX, which is based on the shortest peptide algorithm. For any given residue, the shortest fragment that covers this position was selected and the %D (relative deuterium uptake value) of this peptide was assigned to the residue. The %D was calculated based on the formula,

$$\%D=\frac{m_{t}-m_{0}}{m_{\max}-m_{0}}\times 100,$$

where *m_t_* is the mass of the peptide at exchange time *t*, *m_0_* is the mass of the undeuterated control peptide and *m_max_* is the theoretical maximum mass of the peptide when deuterated (100% deuterated).

Structures were colored based on the B-factor using a blue–white–red gradient color scale from 0 to 50% for individual samples and from −25% to +25% for differential comparisons. Three replicate measurements were performed for each condition. ata are available via ProteomeXchange (56) with identifier PXD86124 (57).

### Turbidity Assay.

Aggregation during spontaneous refolding was monitored by absorbance at 320 nm for 30 min using a V-760 spectrophotometer (Jasco). QM-MBP was denatured in 6 M GuHCl at RT for 1 h. Samples were diluted 200-fold into refolding buffer A to final concentrations of 50, 100, 200, and 500 nM. Native QM-MBP (100 nM) served as negative control, while MetF denatured in 6 M GuHCl served as a positive aggregation control.

### Thermal Stability Analysis by CD Spectroscopy.

The *T*_m_ of wild-type (WT) GroEL (1 µM) in refolding buffer A was determined by monitoring changes in the CD signal at 222 nm while increasing the temperature from 4 to 90 °C at a rate of 1 ^o^C min^−^^1^ using a J-715 spectropolarimeter (Jasco). The transition curves were fitted and calculated by Spectra Manager software (Jasco). Measurements were repeated 3 times and the *T*_m_ values expressed as mean ± SEM.

### Thermal Stability (*T*_m_) Measurements by nanoDSF.

NanoDSF measurements were performed using 1 µM QM-MBP in refolding buffer A containing 1 mM BeFx. Free QM-MBP and free QM-MBP together with empty GroEL:(ES)_2_ (2 µM) complexes served as control for chaperonin-encapsulated QM-MBP (1 µM). GroEL:(ES)_2_ complexes and chaperonin-encapsulated QM-MBP were prepared as described above. Thermal unfolding was monitored by recording fluorescence intensity changes at 350 nm between 20 °C and 90 °C, with the rate of temperature increase set to 5 °C min^−1^ using a Prometheus NT.48 nanoDSF instrument (NanoTemper). *T*_m_ values were automatically calculated by the software PR.ThermControl V2.1.2 (NanoTemper). Measurements were repeated 3 times and the *T*_m_ values expressed as mean ± SEM.

### Reversible Refolding of Chaperonin-Encapsulated QM-MBP(SS) Analyzed by nanoDSF.

GroEL (E257C):ES(G29C)-encapsulated QM-MBP(SS) was prepared using the method described above, except that the refolding buffer E [20 mM MOPS-KOH, pH 6.5, 10 mM Mg(OAC)_2_, 1 mM BeFx], which contains no KCl and a lower pH value, was used to reduce the release of QM-MBP(SS) from the GroEL:ES cage at high temperature. To ensure that the QM-MBP(SS) remains encapsulated during in-cage unfolding, the chaperonin-encapsulated QM-MBP(SS) complex was air-oxidized overnight at RT to induce the formation of disulfide bonds between GroEL(E257C) and GroES(G29C), as well as the intramolecular disulfide bonds in QM-MBP(SS). The complex was subsequently isolated by SEC [Superose 6 Increase 3.2/300 column (Cytiva) equilibrated with refolding buffer E]. Free QM-MBP(SS) (1 µM) was used as a control. The thermal unfolding and refolding was performed in three continuous steps (Fig. 5*E*): 1) unfolding by increasing the temperature from 20 to 60 ^o^C; 2) refolding during temperature downshift from 60 to 20 ^o^C; 3) unfolding during temperature upshift from 20 to 60 ^o^C. Heating and cooling phases were performed at a rate of 1 °C min^−1^. All the measurements were repeated 3 times. *T*_m_ and T*_rf_* (temperature of half-maximal refolding) values were expressed as mean ± SEM.

### Calculation of Gibbs Free Energy of Unfolding.

The change in Gibbs free energy (ΔG°) of unfolding was determined from thermal denaturation curves using the Van’t Hoff analysis. The thermodynamic parameters were calculated using the following equations:

$$\Delta G^{\circ }=\;\Delta H^{\circ }-\;T\Delta S^{\circ }=\;-RT\;\ln K_{\mathrm{eq}},$$

$$K_{\mathrm{eq}}=\frac{f_{D}}{1-f_{D}},$$

$$\text{ln}K_{\mathrm{eq}}=\;-\frac{\Delta H^{\circ }}{R}\cdot \left(\frac{1}{T}\right)+\frac{\Delta S^{\circ }}{R},$$

where *K_eq_* is the equilibrium constant of denaturation, defined as the ratio of the fraction of denatured protein (*f_D_*) to the native protein (1 – *f_D_*). The fraction of denatured protein (*f_D_*) was calculated with the following equation, assuming that the protein is fully folded and unfolded at a temperature of 4 °C below and above *T_m_*, respectively (free QM-MBP as example):

$$f_{D}=\frac{F350_{\left(40^{\circ \text{C}}\right)}-F350_{\left(x^{\circ \text{C}}\right)}}{F350_{\left(40^{\circ \text{C}}\right)}-F350_{\left(48^{\circ \text{C}}\right)}},$$

The *K_eq_* values were derived from the transition region of the denaturation curves for both free and chaperonin-encapsulated QM-MBP. A van’t Hoff plot of ln*K_eq_* versus the reciprocal of temperature (1/*T*) was generated. Linear regression analysis was used to determine the standard enthalpy change (Δ*H*°) from the slope of the fitted line. The standard entropy change (Δ*S*°) was subsequently calculated using the melting temperature (*T*_m_), where Δ*G*° = 0, such that Δ*S*° = Δ*H*° /*T*_m_.

### Refolding Kinetics of QM-MBP.

For spontaneous refolding, QM-MBP was denatured in 6 M GuHCl at RT for 1 h and diluted 200-fold into refolding buffer A to a final concentration of 100 nM (or 50 to 200 nM to assay concentration dependence). For chaperonin-rescue of refolding, 1 µM GroEL, 2 µM GroES, and 5 mM ATP were added to the spontaneous refolding reaction at specific time points (0, 30, or 60 min) after initiating spontaneous refolding. Folding kinetics were monitored via tryptophan fluorescence (excitation, λ_ex_, at 295 nm and emission, λ_em_, at 345 nm) on a Fluorolog-2 spectrofluorometer (Horiba). Signals were recorded every 30 s for 3 h (rescued) or 20 h (spontaneous). All measurements were repeated 3 times and each point is shown as mean ± SEM. The t_1/2_ values were obtained from curve fits in Origin.

### Analysis of GroES Exchange in GroEL:ES Complexes.

4 µM Alexa-555 labeled GroES(98C) was added to 2 µM GroEL:(ES)_2_:ADP^.^BeFx with encapsulated QM-MBP or DM-MBP in refolding buffer A containing 1 mM BeFx, followed by incubation at different temperatures (20, 30, 40, 45, 50, 55, 60 ^o^C) for 3 min and subsequent cooling to 20 °C for 10 min. The reactions were subjected to SEC [Superose 6 Increase 3.2/300 column (Cytiva) equilibrated in refolding buffer A containing 1 mM BeFx] to separate free from GroEL-bound GroES. The SEC fractions were analyzed by SDS-PAGE, Coomassie blue staining and fluorescence imaging (λ_ex_ at 555 nm and λ_em_ 565 nm; Amersham Typhoon imager, Cytiva). The ratio of GroES exchange was calculated based on the ratio of fluorescence intensity of labeled GroES in the GroEL-containing fractions compared to the total in the fluorescence image (normalized to the maximum observed exchanging ratio).

## Supplementary Material

Appendix 01 (PDF)

## Data Availability

The raw hydrogen/deuterium exchange mass spectrometry data have been deposited to the ProteomeXchange Consortium via the PRIDE (58) partner repository with the dataset identifier PXD086124 (57). All other data are included in the article and/or *SI Appendix*.

## References

[1] T. Lopez, K. Dalton, J. Frydman, The mechanism and function of group II chaperonins. J. Mol. Biol. **427**, 2919–2930 (2015). 10.1016/j.jmb.2015.04.013 PMC4706738 25936650

[2] Y. Cho , Individual and collective contributions of chaperoning and degradation to protein homeostasis in E. coli. Cell Rep. **11**, 321–333 (2015). 10.1016/j.celrep.2015.03.018 PMC4401642 25843722

[3] M. Hayer-Hartl, A. Bracher, F. U. Hartl, The GroEL-GroES chaperonin machine: A nano-cage for protein folding. Trends Biochem. Sci. **41**, 62–76 (2016). 10.1016/j.tibs.2015.07.009 26422689

[4] D. Balchin, M. Hayer-Hartl, F. U. Hartl, In vivo aspects of protein folding and quality control. Science **353**, aac4354 (2016). 10.1126/science.aac4354 27365453

[5] A. L. Horwich, W. A. Fenton, Chaperonin-assisted protein folding: A chronologue. Q. Rev. Biophys. **53**, e4 (2020). 10.1017/S0033583519000143 32070442

[6] E. T. Powers, L. M. Gierasch, The proteome folding problem and cellular proteostasis. J. Mol. Biol. **433**, 167197 (2021). 10.1016/j.jmb.2021.167197 PMC8502207 34391802

[7] H. Saibil, Chaperone machines for protein folding, unfolding and disaggregation. Nat. Rev. Mol. Cell Biol. **14**, 630–642 (2013). 10.1038/nrm3658 PMC4340576 24026055

[8] G. Stan, G. H. Lorimer, D. Thirumalai, Friends in need: How chaperonins recognize and remodel proteins that require folding assistance. Front. Mol. Biosci. **9**, 1071168 (2022). 10.3389/fmolb.2022.1071168 PMC9720267 36479385

[9] A. Horovitz, K. R. Willison, Allosteric regulation of chaperonins. Curr. Opin. Struct. Biol. **15**, 646–651 (2005). 10.1016/j.sbi.2005.10.001 16249079

[10] O. Fayet, T. Ziegelhoffer, C. Georgopoulos, The groES and groEL heat shock gene products of Escherichia coli are essential for bacterial growth at all temperatures. J. Bacteriol. **171**, 1379–1385 (1989). 10.1128/jb.171.3.1379-1385.1989 PMC209756 2563997

[11] S. Guyot, L. Pottier, E. Ferret, L. Gal, P. Gervais, Physiological responses of Escherichia coli exposed to different heat-stress kinetics. Arch. Microbiol. **192**, 651–661 (2010). 10.1007/s00203-010-0597-1 20549191

[12] J. Wagner , Visualizing chaperonin function in situ by cryo-electron tomography. Nature **633**, 459–464 (2024). 10.1038/s41586-024-07843-w PMC11390479 39169181

[13] A. Brinker , Dual function of protein confinement in chaperonin-assisted protein folding. Cell **107**, 223–233 (2001). 10.1016/s0092-8674(01)00517-7 11672529

[14] Y. C. Tang , Structural features of the GroEL-GroES nano-cage required for rapid folding of encapsulated protein. Cell **125**, 903–914 (2006). 10.1016/j.cell.2006.04.027 16751100

[15] K. Chakraborty , Chaperonin-catalyzed rescue of kinetically trapped states in protein folding. Cell **142**, 112–122 (2010). 10.1016/j.cell.2010.05.027 20603018

[16] A. L. Mallam, S. E. Jackson, Knot formation in newly translated proteins is spontaneous and accelerated by chaperonins. Nat. Chem. Biol. **8**, 147–153 (2011). 10.1038/nchembio.742 22179065

[17] A. J. Gupta, S. Haldar, G. Miličić, F. U. Hartl, M. Hayer-Hartl, Active cage mechanism of chaperonin-assisted protein folding demonstrated at single-molecule level. J. Mol. Biol. **426**, 2739–2754 (2014). 10.1016/j.jmb.2014.04.018 24816391

[18] F. Georgescauld , GroEL/ES chaperonin modulates the mechanism and accelerates the rate of TIM-barrel domain folding. Cell **157**, 922–934 (2014). 10.1016/j.cell.2014.03.038 PMC4071350 24813614

[19] M. A. Soler, A. Rey, P. F. Faísca, Steric confinement and enhanced local flexibility assist knotting in simple models of protein folding. Phys. Chem. Chem. Phys. **18**, 26391–26403 (2016). 10.1039/c6cp05086g 27722468

[20] J. Weaver , GroEL actively stimulates folding of the endogenous substrate protein PepQ. Nat. Commun. **8**, 15934 (2017). 10.1038/ncomms15934 PMC5497066 28665408

[21] A. K. Singh, D. Balchin, R. Imamoglu, M. Hayer-Hartl, F. U. Hartl, Efficient catalysis of protein folding by GroEL/ES of the obligate chaperonin substrate MetF. J. Mol. Biol. **432**, 2304–2318 (2020). 10.1016/j.jmb.2020.02.031 32135190

[22] M. M. Naqvi , Protein chain collapse modulation and folding stimulation by GroEL-ES. Sci. Adv. **8**, eabl6293 (2022). 10.1126/sciadv.abl6293 PMC8896798 35245117

[23] G. Agam, A. Barth, D. C. Lamb, Folding pathway of a discontinuous two-domain protein. Nat. Commun. **15**, 690 (2024). 10.1038/s41467-024-44901-3 PMC10805907 38263337

[24] D. H. Chen , Visualizing GroEL/ES in the act of encapsulating a folding protein. Cell **153**, 1354–1365 (2013). 10.1016/j.cell.2013.04.052 PMC3695626 23746846

[25] S. Sharma , Monitoring protein conformation along the pathway of chaperonin-assisted folding. Cell **133**, 142–153 (2008). 10.1016/j.cell.2008.01.048 18394994

[26] J. L. England, V. S. Pande, Potential for modulation of the hydrophobic effect inside chaperonins. Biophys. J. **95**, 3391–3399 (2008). 10.1529/biophysj.108.131037 PMC2547425 18599630

[27] J. L. England, D. Lucent, V. S. Pande, A role for confined water in chaperonin function. J. Am. Chem. Soc. **130**, 11838–11839 (2008). 10.1021/ja802248m PMC2646679 18710231

[28] I. Korobko, H. Mazal, G. Haran, A. Horovitz, Measuring protein stability in the GroEL chaperonin cage reveals massive destabilization. eLife **9**, e56511 (2020). 10.7554/eLife.56511 PMC7440923 32716842

[29] I. Korobko, R. B. Eberle, M. Roy, A. Horovitz, A diminished hydrophobic effect inside the GroEL/ES cavity contributes to protein substrate destabilization. Proc. Natl. Acad. Sci. U.S.A. **119**, e2213170119 (2022). 10.1073/pnas.2213170119 PMC9860310 36409898

[30] W. J. Becktel, J. A. Schellman, Protein stability curves. Biopolymers **26**, 1859–1877 (1987). 10.1002/bip.360261104 3689874

[31] M. K. Hayer-Hartl, F. U. Hartl, A comment on:’The aromatic amino acid content of the bacterial chaperone protein groEL (cpn60): evidence for the presence of a single tryptophan’, by N.C. Price, S.M. Kelly, S. Wood and A. auf der Mauer (1991) FEBS Lett. 292, 9-12. FEBS Lett. **320**, 83–84 (1993). 10.1016/0014-5793(91)80821-j 1683633

[32] O. Boudker, M. J. Todd, E. Freire, The structural stability of the co-chaperonin GroES. J. Mol. Biol. **272**, 770–779 (1997). 10.1006/jmbi.1997.1263 9368656

[33] B. Sot, S. Bañuelos, J. M. Valpuesta, A. Muga, GroEL stability and function. Contribution of the ionic interactions at the inter-ring contact sites. J. Biol. Chem. **278**, 32083–32090 (2003). 10.1074/jbc.M303958200 12796493

[34] A. Koike-Takeshita, M. Yoshida, H. Taguchi, Revisiting the GroEL-GroES reaction cycle via the symmetric intermediate implied by novel aspects of the GroEL(D398A) mutant. J. Biol. Chem. **283**, 23774–23781 (2008). 10.1074/jbc.M802542200 PMC3259786 18567584

[35] H. Taguchi, K. Tsukuda, F. Motojima, A. Koike-Takeshita, M. Yoshida, BeF(x) stops the chaperonin cycle of GroEL-GroES and generates a complex with double folding chambers. J. Biol. Chem. **279**, 45737–45743 (2004). 10.1074/jbc.M406795200 15347650

[36] X. Fei, X. Ye, N. A. LaRonde, G. H. Lorimer, Formation and structures of GroEL:GroES2 chaperonin footballs, the protein-folding functional form. Proc. Natl. Acad. Sci. U.S.A. **111**, 12775–12780 (2014). 10.1073/pnas.1412922111 PMC4156775 25136110

[37] A. Koike-Takeshita, T. Arakawa, H. Taguchi, T. Shimamura, Crystal structure of a symmetric football-shaped GroEL:GroES(2)-ATP(14) complex determined at 3.8 angstrom reveals rearrangement between two GroEL Rings. J. Mol. Biol. **426**, 3634–3641 (2014). 10.1016/j.jmb.2014.08.017 25174333

[38] X. Yan , GroEL ring separation and exchange in the chaperonin reaction. Cell **172**, 605–617.e611 (2018). 10.1016/j.cell.2017.12.010 29336887

[39] Y. Zhang, C. Conway, M. Rosato, Y. Suh, M. D. Manson, Maltose chemotaxis involves residues in the N-terminal and C-terminal domains on the same face of maltose-binding protein. J. Biol. Chem. **267**, 22813–22820 (1992). 1429629

[40] M. de Boer , Conformational and dynamic plasticity in substrate-binding proteins underlies selective transport in ABC importers. eLife **8**, e44652 (2019). 10.7554/eLife.44652 PMC6450668 30900991

[41] M. van den Noort, M. de Boer, B. Poolman, Stability of ligand-induced protein conformation influences affinity in maltose-binding protein. J. Mol. Biol. **433**, 167036 (2021). 10.1016/j.jmb.2021.167036 33957147

[42] A. N. Kapanidis , Fluorescence-aided molecule sorting: Analysis of structure and interactions by alternating-laser excitation of single molecules. Proc. Natl. Acad. Sci. U.S.A. **101**, 8936–8941 (2004). 10.1073/pnas.0401690101 PMC428450 15175430

[43] B. K. Müller, E. Zaychikov, C. Bräuchle, D. C. Lamb, Pulsed interleaved excitation. Biophys. J. **89**, 3508–3522 (2005). 10.1529/biophysj.105.064766 PMC1366845 16113120

[44] G. Agam , Reliability and accuracy of single-molecule FRET studies for characterization of structural dynamics and distances in proteins. Nat. Methods **20**, 523–535 (2023). 10.1038/s41592-023-01807-0 PMC10089922 36973549

[45] E. I. James, T. A. Murphree, C. Vorauer, J. R. Engen, M. Guttman, Advances in hydrogen/deuterium exchange mass spectrometry and the pursuit of challenging biological systems. Chem. Rev. **122**, 7562–7623 (2022). 10.1021/acs.chemrev.1c00279 PMC9053315 34493042

[46] D. B. Craig, A. A. Dombkowski, Disulfide by design 2.0: A web-based tool for disulfide engineering in proteins. BMC Bioinformatics **14**, 346 (2013). 10.1186/1471-2105-14-346 PMC3898251 24289175

[47] D. S. Libich, V. Tugarinov, R. Ghirlando, G. M. Clore, Confinement and stabilization of Fyn SH3 folding intermediate mimetics within the cavity of the chaperonin GroEL demonstrated by relaxation-based NMR. Biochemistry **56**, 903–906 (2017). 10.1021/acs.biochem.6b01237 PMC5404735 28156097

[48] Y. Xu, G. Feller, C. Gerday, N. Glansdorff, *Moritella* cold-active dihydrofolate reductase: Are there natural limits to optimization of catalytic efficiency at low temperature? J. Bacteriol. **185**, 5519–5526 (2003). 10.1128/JB.185.18.5519-5526.2003 PMC193739 12949104

[49] R. Horst, W. A. Fenton, S. W. Englander, K. Wüthrich, A. L. Horwich, Folding trajectories of human dihydrofolate reductase inside the GroEL GroES chaperonin cavity and free in solution. Proc. Natl. Acad. Sci. U.S.A. **104**, 20788–20792 (2007). 10.1073/pnas.0710042105 PMC2410080 18093916

[50] A. Roeselová , GroEL/ES chaperonin unfolds then encapsulates a nascent protein on the ribosome. Nat. Commun. **16**, 9976 (2025). 10.1038/s41467-025-64968-w PMC12615815 41233324

[51] M. K. Hayer-Hartl, F. Weber, F. U. Hartl, Mechanism of chaperonin action: GroES binding and release can drive GroEL-mediated protein folding in the absence of ATP hydrolysis. EMBO J. **15**, 6111–6121 (1996). PMC452432 8947033

[52] M. K. Hayer-Hartl, J. J. Ewbank, T. E. Creighton, F. U. Hartl, Conformational specificity of the chaperonin GroEL for the compact folding intermediates of alpha-lactalbumin. EMBO J. **13**, 3192–3202 (1994). 10.1002/j.1460-2075.1994.tb06618.x PMC395211 7913682

[53] R. Imamoglu, D. Balchin, M. Hayer-Hartl, F. U. Hartl, Bacterial Hsp70 resolves misfolded states and accelerates productive folding of a multi-domain protein. Nat. Commun. **11**, 365 (2020). 10.1038/s41467-019-14245-4 PMC6969021 31953415

[54] A. A. Deniz , Single-pair fluorescence resonance energy transfer on freely diffusing molecules: Observation of Förster distance dependence and subpopulations. Proc. Natl. Acad. Sci. U.S.A. **96**, 3670–3675 (1999). 10.1073/pnas.96.7.3670 PMC22352 10097095

[55] D. Balchin, G. Miličić, M. Strauss, M. Hayer-Hartl, F. U. Hartl, Pathway of actin folding directed by the eukaryotic chaperonin TRiC. Cell **174**, 1507–1521.e16 (2018). 10.1016/j.cell.2018.07.006 30100183

[56] E. W. Deutsch ., The ProteomeXchange consortium in 2026: Making proteomics data FAIR. *Nucleic Acids Res.* **54**, D459-D469 (2026). 10.1093/nar/gkaf1146 PMC12807779 41206473

[57] W. Xue, A. Bracher, F.U. Hartl, M. Hayer-Hartl, Data from “Thermodynamic stabilization of substrate protein within the GroEL:ES chaperonin cage.” PRIDE Database. https://www.ebi.ac.uk/pride/archive/projects/PXD086124. Deposited 22 October 2026. 10.1073/pnas.2624029123 PMC13751682 42975935

[58] Y. Perez-Riverol ., The PRIDE database at 20 years: 2025 update. *Nucleic Acids Res.* **53**, D543-D553 (2025). 10.1093/nar/gkae1011 PMC11701690 39494541

