# Supplementary material for: Thermodynamic stabilization of substrate protein within the GroEL:ES chaperonin cage
Source: Proc Natl Acad Sci U S A. 2026 Nov 3;123(45):e2624029123. doi: 10.1073/pnas.2624029123 (PMC13751682; doi:10.1073/pnas.2624029123)
Supplement: Supplementary file 1 [file pnas.2624029123.sapp.pdf]

Supporting Information for

Thermodynamic stabilization of substrate protein within the GroEL:ES chaperonin cage

Wei Xue, Andreas Bracher, F. Ulrich Hartl and Manajit Hayer-Hartl

F. Ulrich Hartl and Manajit Hayer-Hartl
Email: uhartl@biochem.mpg.de; mhartl@biochem.mpg.de

This PDF file includes:

Figures S1 to S6

Figures

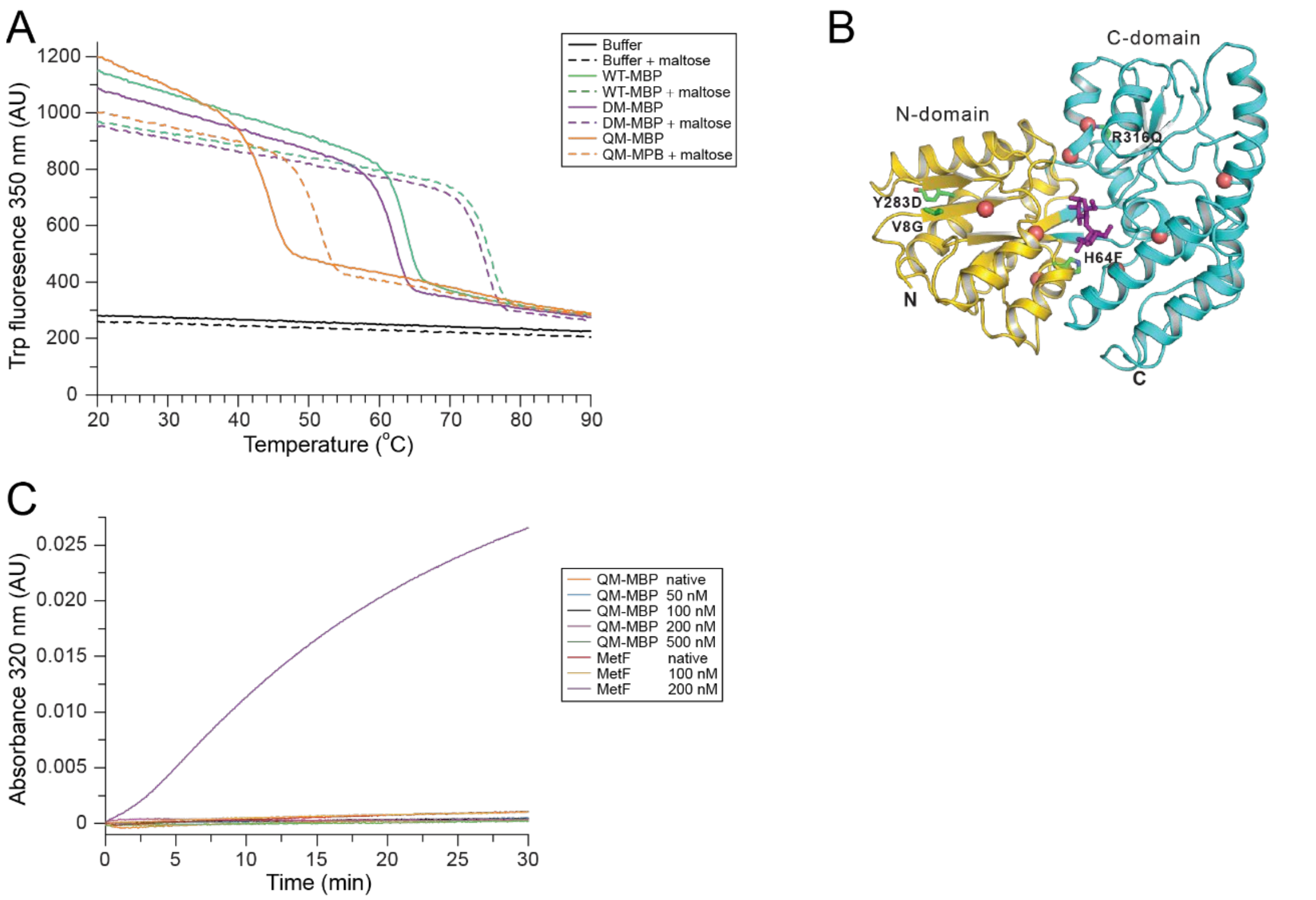

Fig. S1. Folding and stability of QM-MBP. (*A*) Representative melting curves for WT-, DM-, and QM-MBP (1 µM) in refolding buffer A in presence or absence of 10 mM maltose. T_m values were determined by monitoring tryptophane fluorescence intensity changes at 350 nm over a temperature range of 20-90 °C by nano-DSF. (*B*) Structural model of maltose-bound MBP (PDB: 1ANF). Maltose-bound MBP is shown in ribbon representation as in Fig. 1B. The bound maltose (purple) and the residue substitutions in QM-MBP are shown in stick representation. Tryptophan (Trp) residues are indicated as spheres (salmon). N- and C-terminus are indicated. (*C*) Turbidity at increasing concentrations (50, 100, 200, 500 nM) was monitored during spontaneous refolding of QM-MBP by absorbance at 320 nm for 30 min in refolding buffer A. 100 nM native QM-MBP was used as negative control and spontaneously refolding MetF at concentrations of 100 and 200 nM served as positive control.

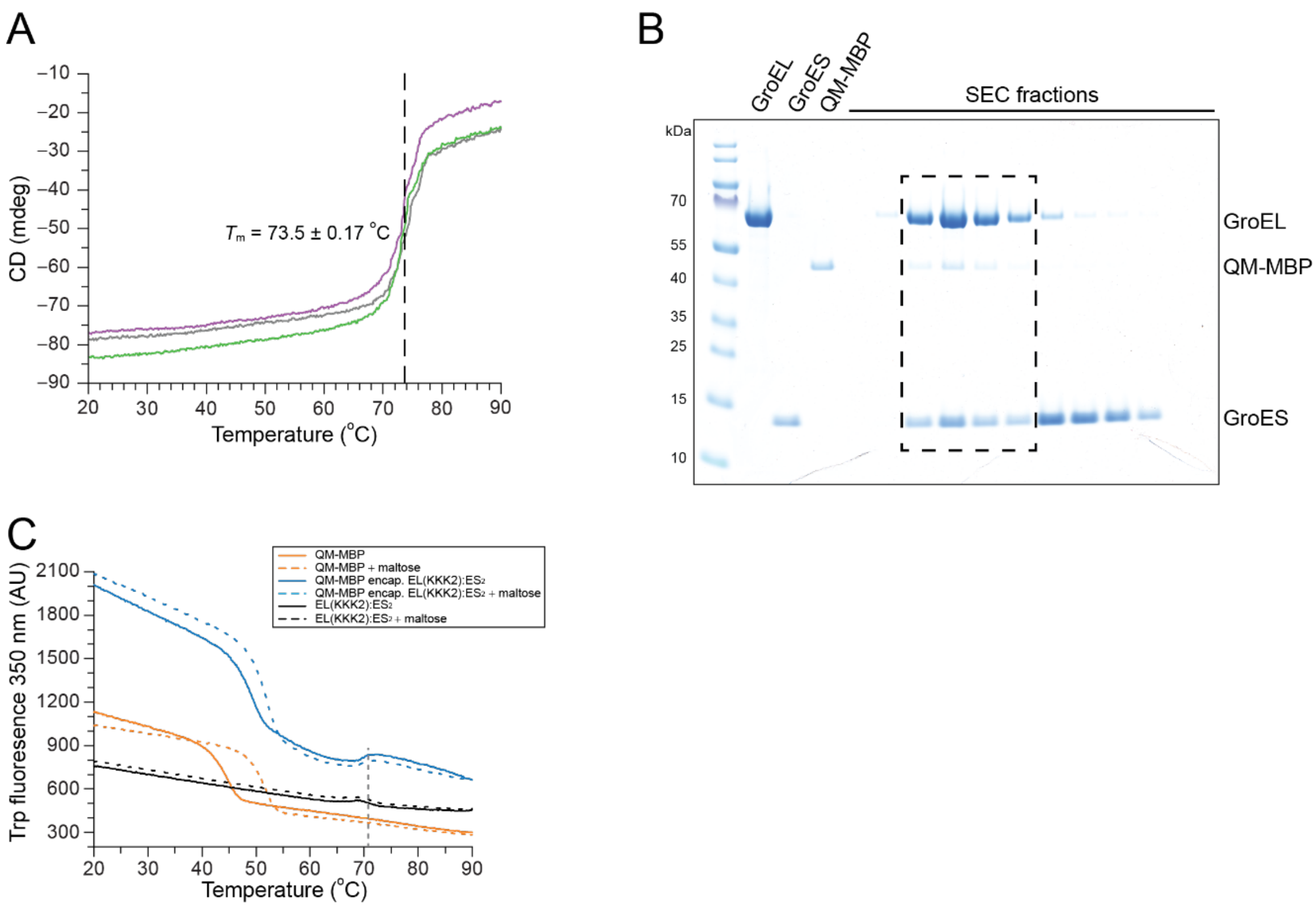

Fig. S2. Stability of chaperonin-encapsulated QM-MBP. (*A*) Melting curves of WT-GroEL (1 μM) measured by CD spectroscopy. The CD signal of WT-GroEL at 222 nm was monitored from 4 to 90 °C using a heating rate of 1 °C min^{-1} in refolding buffer A. The vertical dashed line shows the T_m value of WT-GroEL (n=3, mean ± SEM). (*B*) SDS-PAGE of SEC fractions of chaperonin-encapsulated QM-MBP. (*C*) Thermal denaturation of EL(KKK2):ES_2 with 1 μM encapsulated QM-MBP. Melting curves were recorded by nanoDSF at 350 nm emission wavelength over the temperature range of 20 to 90 °C in refolding buffer A containing 1 mM BeFx in the presence or absence of maltose (10 mM). For comparison, melting curves of free QM-MBP in presence or absence of chaperonin complex and chaperonin complex alone are also shown. Representative melting curves are shown (n=3).

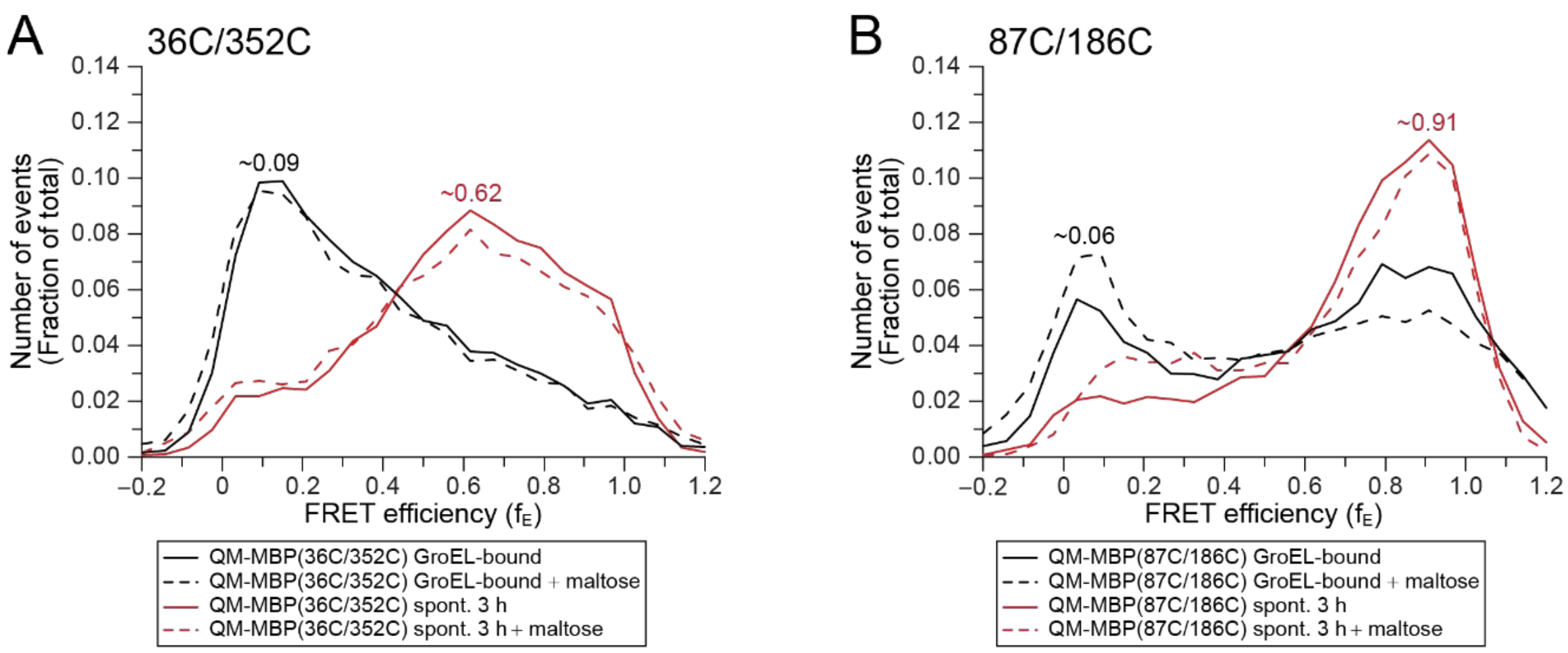

Fig. S3. Probing the compaction of QM-MBP by single molecule fluorescence resonance energy transfer (smFRET). (*A* and *B*) FRET efficiency (f_E) histograms for labeled QM-MBP(T36C/S352C) (*A*) and QM-MBP(D87C/A186C) (*B*). GroEL-bound QM-MBP and QM-MBP kinetically trapped during 3 h of spontaneous refolding at 100 pM in FRET buffer C in presence or absence of maltose (10 mM) was analyzed by smFRET. For spontaneous refolding, 20 nM QM-MBP was denatured in 6 M GuHCl and diluted 200-fold into FRET buffer (see Methods). smFRET measurements were taken after 3 h of refolding for a period of 15 min (see Methods). f_E distributions in presence of maltose are indicated by dashed lines.

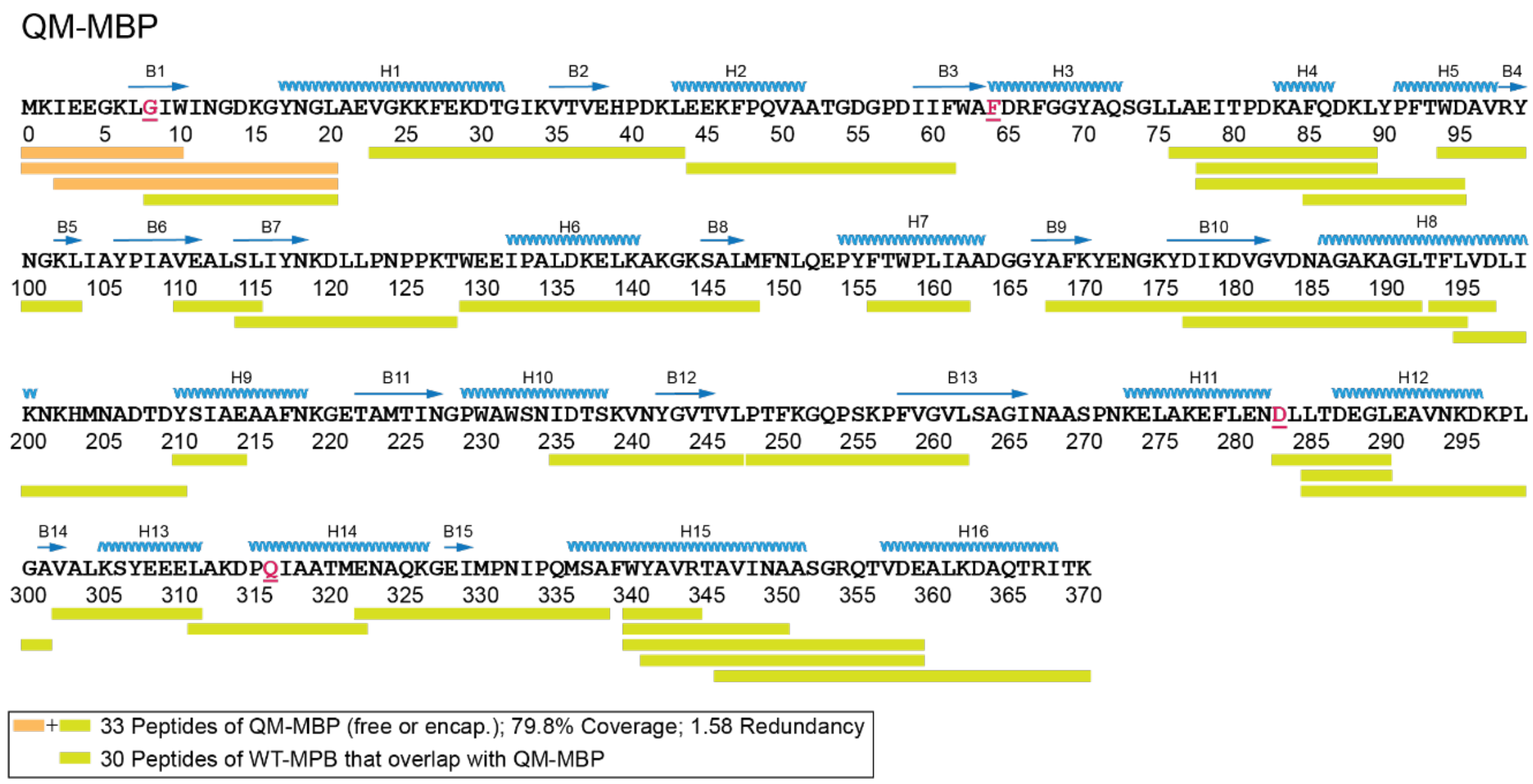

Fig. S4. HD/X analysis of QM- and WT-MBP at peptide resolution in the absence or presence of maltose. The 4 residues mutated in QM-MBP are indicated in red. The secondary structure elements are shown above the MBP sequence. The bars below the sequence indicate the peptides identified, covering ~80 % of the MBP sequence, including 33 peptides of free or chaperonin-encapsulated QM-MBP and 30 peptides overlapping with WT-MBP. For the 30 overlapping peptides, the peptides 8-20, 283-290 and 311-322 each have a mutated residue in QM-MBP and thus only 27 peptides were included for the H/DX structural comparison (Fig. 4*B*). Green bars, peptides identified in QM- and WT-MBP; Orange bars, peptides identified only in QM-MBP.

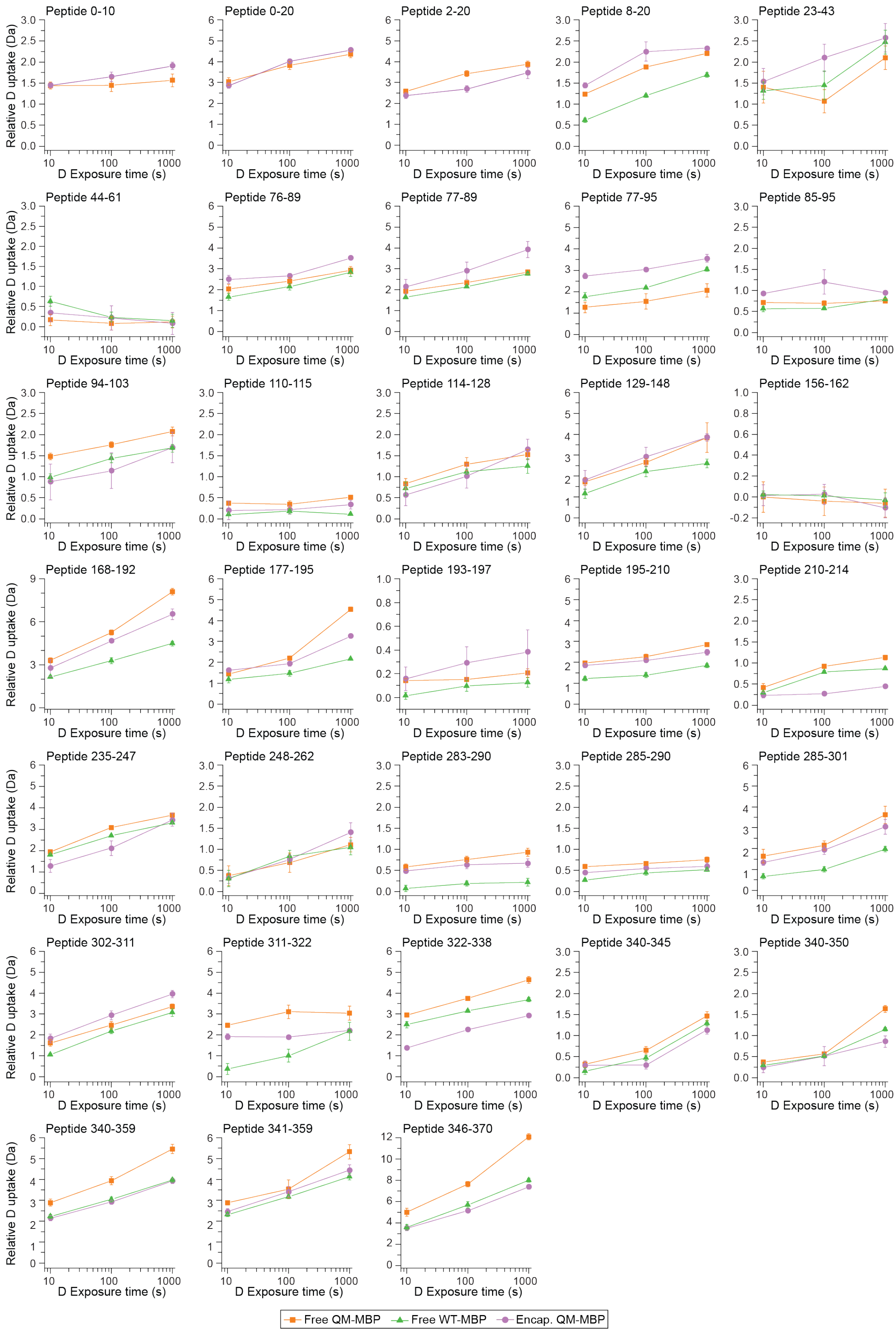
Peptide 0-10
Peptide 0-20
Peptide 2-20
Peptide 8-20
Peptide 23-43
Peptide 44-61
Peptide 76-89
Peptide 77-89
Peptide 77-95
Peptide 85-95
Peptide 94-103
Peptide 110-115
Peptide 114-128
Peptide 129-148
Peptide 156-162
Peptide 168-192
Peptide 177-195
Peptide 193-197
Peptide 195-210
Peptide 210-214
Peptide 235-247
Peptide 248-262
Peptide 283-290
Peptide 285-290
Peptide 285-301
Peptide 302-311
Peptide 311-322
Peptide 322-338
Peptide 340-345
Peptide 340-350
Peptide 340-359
Peptide 341-359
Peptide 346-370
Relative D uptake (Da)
D Exposure time (s)
Free QM-MBP
Free WT-MBP
Encap. QM-MBP

Fig. S5. Time-resolved deuterium incorporation plots in the absence of maltose for the 33 peptides of free or chaperonin-encapsulated QM-MBP and 30 peptides of WT-MBP. Data are shown for free QM-MBP (orange), free WT-MBP (green), and chaperonin-encapsulated QM-MBP (purple). In QM-MBP the peptides 8-20, 283-290 and 311-322 each have a mutated residue compared to WT-MBP. Error bars represent SD (n=3).

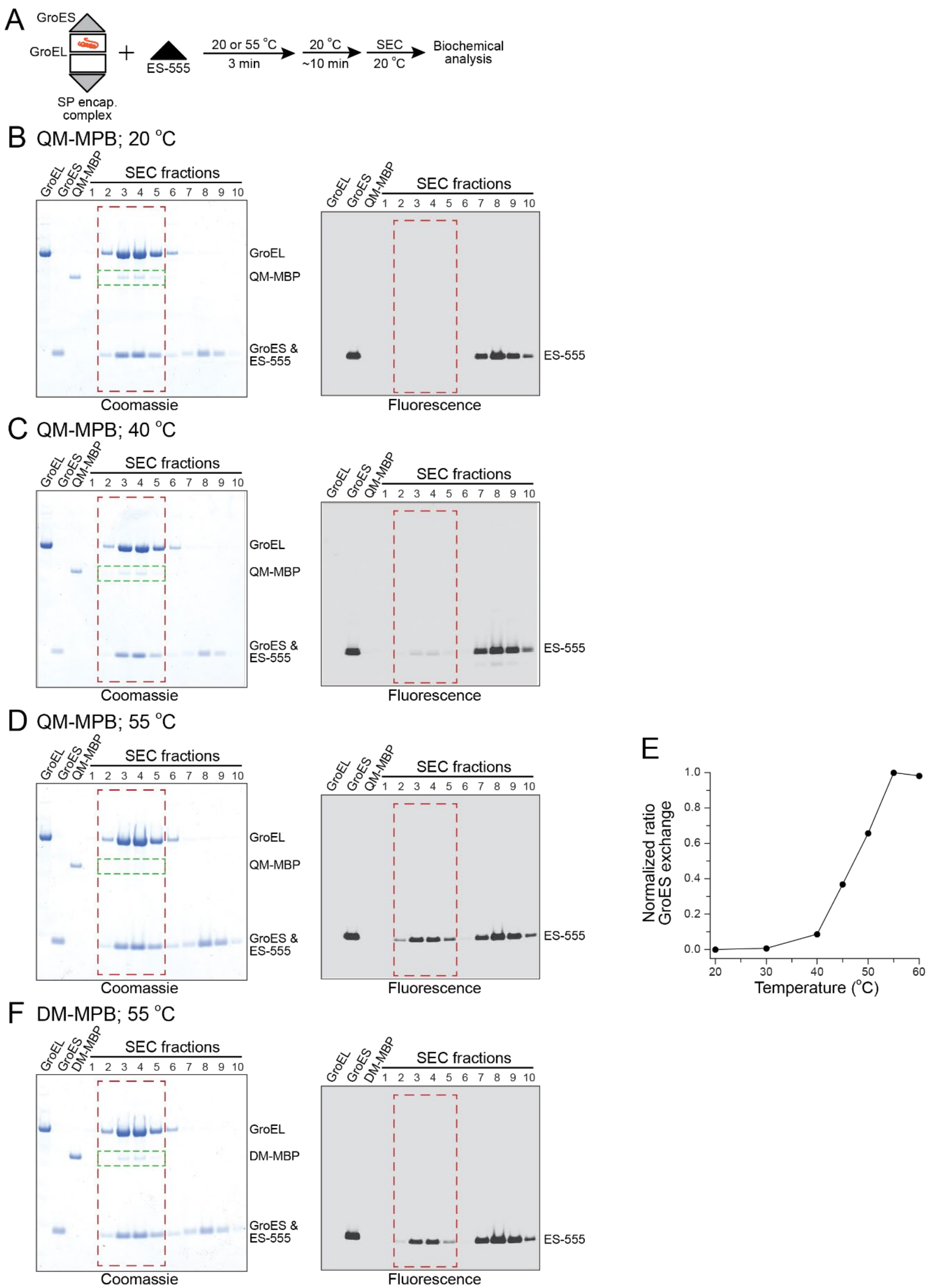
A
GroES
GroEL
SP encap. complex
ES-555
20 or 55 °C
3 min
20 °C
~10 min
SEC
20 °C
Biochemical analysis
B QM-MPB; 20 °C
C QM-MPB; 40 °C
D QM-MPB; 55 °C
F DM-MPB; 55 °C
GroEL
GroES
QM-MBP
DM-MBP
SEC fractions
1 2 3 4 5 6 7 8 9 10
GroES & ES-555
ES-555
Coomassie
Fluorescence
E
Normalized ratio GroES exchange
Temperature (°C)
0.0 0.2 0.4 0.6 0.8 1.0
20 30 40 50 60

Fig. S6. Temperature dependent GroES exchange. (*A*) Schematic depicting the analysis of exchange of GroEL-bound GroES with free, fluorescent labeled GroES. Alexa-555 labeled GroES(98C) (4 μM) was incubated with preformed complexes of chaperonin-encapsulated QM-MBP or DM-MBP (2 μM complex, based on GroEL concentration) in refolding buffer A containing 1 mM BeFx at increasing temperatures (20 °C-60 °C) for 3 min, followed by incubation at 20 °C for 10 min. Reactions were then separated by SEC and fractions analyzed by SDS-PAGE with Coomassie blue staining and fluorescence imaging. (*B*-*D*) SDS-PAGE of SEC fractions of chaperonin-encapsulated QM-MBP at 20 °C *(B*), 40 °C *(C*) and 55°C (*D*). Coomassie blue staining and fluorescence images are shown on the left and right panels, respectively. The red and green dashed boxes indicate the fractions containing GroEL:ES complexes and encapsulated QM-MBP, respectively. (*E*) Temperature dependence of GroES exchange shown as the normalized ratio of ES-555 in GroEL:ES containing fractions (red boxed area in (*B*-*D*)) to the total ES-555. (*F*) SDS-PAGE of the SEC fractions for encapsulated DM-MBP at 55°C, analyzed and displayed as in (*B*-*D*).
